# Contribution of Individual Ebp Pilus Subunits of *Enterococcus faecalis* OG1RF to Pilus Biogenesis, Biofilm Formation and Urinary Tract Infection

**DOI:** 10.1371/journal.pone.0068813

**Published:** 2013-07-11

**Authors:** Jouko Sillanpää, Chungyu Chang, Kavindra V. Singh, Maria Camila Montealegre, Sreedhar R. Nallapareddy, Barrett R. Harvey, Hung Ton-That, Barbara E. Murray

**Affiliations:** 1 Division of Infectious Diseases, Department of Internal Medicine, University of Texas Medical School, Houston, Texas, United States of America; 2 Center for the Study of Emerging and Re-emerging Pathogens, University of Texas Medical School, Houston, Texas, United States of America; 3 Department of Microbiology and Molecular Genetics, University of Texas Medical School, Houston, Texas, United States of America; 4 Center for Immunology and Autoimmune Diseases, Brown Foundation Institute of Molecular Medicine for the Prevention of Human Diseases, University of Texas Medical School, Houston, Texas, United States of America; Kansas State University, United States of America

## Abstract

The endocarditis and biofilm-associated pilus (Ebp) operon is a component of the core genome of *Enterococcus faecalis* that has been shown to be important for biofilm formation, adherence to host fibrinogen, collagen and platelets, and in experimental endocarditis and urinary tract infection models. Here, we created single and double deletion mutants of the pilus subunits and sortases; next, by combining western blotting, immunoelectron microscopy, and using *ebpR in trans* to increase pilus production, we identified EbpA as the tip pilin and EbpB as anchor at the pilus base, the latter attached to cell wall by the housekeeping sortase, SrtA. We also confirmed EbpC and Bps as the major pilin and pilin-specific sortase, respectively, both required for pilus polymerization. Interestingly, pilus length was increased and the number of pili decreased by deleting *ebpA*, while control overexpression of *ebpA in trans* restored wild-type levels, suggesting a dual role for EbpA in both initiation and termination of pilus polymerization. We next investigated the contribution of each pilin subunit to biofilm formation and UTI. Significant reduction in biofilm formation was observed with deletion of *ebpA* or *ebpC* (*P*<0.001) while *ebpB* was found to be dispensable; a similar result was seen in kidney CFUs in experimental UTI (Δ*ebpA*, Δ*ebpC*, *P*≤0.0093; Δ*ebpB*, non-significant, each vs. OG1RF). Hence, our data provide important structural and functional information about these ubiquitous *E. faecalis* pili and, based on their demonstrated importance in biofilm and infection, suggest EbpA and EbpC as potential targets for antibody-based therapeutic approaches.

## Introduction

*Enterococcus faecalis* is a gram-positive coccus that is a common commensal of the human gastrointestinal tract. Although long known for its potential to cause occasional cases of endocarditis, intraabdominal, pelvic and urinary tract infections (UTI) in the community, this organism has been found as the causative agent in a wide variety of nosocomial infections since the mid–late 1970’s, including catheter-associated bacteremia, meningitis and prosthetic device infections, in addition to nosocomial UTIs, intraabdominal infections and endocarditis [1–3].

Our previous studies demonstrated that a pilus-deficient disruption mutant of *E. faecalis* OG1RF was attenuated in a rat endocarditis model and in the ability to form biofilm, leading to the name “endocarditis and biofilm-associated pili” or Ebp [4]. Recently, pilus-deficient mutants were found to be attenuated in catheter-associated and non-catheter associated UTI models [5,6]. Furthermore, Ebp pili are involved in adherence of OG1RF to host fibrinogen and collagen [6] as well as to human platelets [7], thus suggesting that they exhibit multiple functions during the infection process. Ebp pili are encoded by a locus which consists of three structural pilin genes, *ebpA, ebpB* and *ebpC*, and an adjacent downstream gene, *bps* (previously called *srtC*), encoding a class C sortase [4]. Gene expression studies demonstrated that the three *ebp* genes and *bps* produce a single polycistronic transcript and that *bps* is also independently expressed from a second promoter. Unlike pilus loci in streptococci, which are located on genomic islands [8], the *ebp* genes are part of the core genome of this species, and are also very highly conserved across clonal complexes by sequence analyses [4,7]. Only one other pilus, Bee (biofilm enhancer in Enterococcus) [9], carried on a plasmid, has been identified and it is only found in ~1-2% of *E. faecalis* isolates [7].

We previously showed high titers of antibodies to the Ebp subunit proteins in sera from patients with a prior *E. faecalis* infection [10], indicating *in vivo* Ebp expression. More recently, we found increased Ebp amounts on *E. faecalis* OG1RF cells analyzed directly from rat IE vegetations versus *in vitro* grown organisms, demonstrating that Ebp pili are actively expressed in the host during infection [7]. These studies also revealed that Ebp production by OG1RF is positively affected *in vitro* by environmental conditions that may mimic physiologic conditions in the host, i.e., growth in medium (brain heart infusion, BHI) supplemented with serum [4] or bicarbonate [11]. While factors involved in the regulation of Ebp pilus expression are not yet fully understood, studies have identified that *ebpR*, located upstream of *ebpABC* [12], and *rnjB* [13], located elsewhere on the chromosome, both function as positive regulators of *ebp* operon gene expression, while the *fsr* system was shown to be a weak repressor of the *ebpR-ebpABC* locus [11,14].

Nielsen et al. recently showed that the metal ion-dependent adhesion site (MIDAS) motif present on EbpA is important for pilus function and that deletion of *ebpA* altered pilus biogenesis and led to attenuation in an experimental catheter-associated (CA) UTI model [5]. Nonetheless, many questions about how Ebp pili are assembled and the subunit architecture within pilus fibers remain unanswered. Pilus subunit proteins of gram-positive bacteria contain a C-terminal cell-wall sorting signal that includes an LPXTG-like motif. Studies with pili of *Corynebacterium diphtheriae* have led to a pilus assembly model in which a class C sortase [15], encoded within the same pilus gene cluster, catalyzes transpeptidation between the threonine residue of the LPXTG motif and a lysine residue of another pilus subunit (typically within a conserved pilin motif), thus covalently linking the two subunits and leading to successive incorporation of additional subunits [16–18]. Class A sortases [15], also called housekeeping sortases, are generally not located within pilin gene clusters and have a much larger set of substrates, which they attach through their LPXTG motifs to a lipid II peptidoglycan precursor, leading to covalent anchoring of the protein to the cell wall [19]. Pili of *C. diphtheriae*, *Streptococcus agalactiae*, *Streptococcus pyogenes* and *Bacillus cereus* have also been shown to be cell-wall anchored primarily by the housekeeping sortase [20–23]. However, SrtA of *Streptococcus pneumoniae* was found dispensable for localization of pili to the cell wall, suggesting that the housekeeping sortase might not have a universally required role among gram-positive bacteria in pilus attachment to the cell wall [24].

Here, to further investigate the subunit architecture of Ebp pili, we have constructed a series of single and double deletions of all three Ebp pilin-encoding genes and the two sortase-encoding genes of *E. faecalis* OG1RF and investigated their roles in Ebp pilus formation using highly specific antibodies. Finally, the relative importance of each pilin was evaluated for their role in biofilm formation and infection in a mouse non-catheter-associated UTI model. 

## Materials and Methods

### Ethics statement

The mouse urinary tract infection model and surgical procedures were performed in strict accordance with the recommendations in the Guide for the Care and Use of Laboratory Animals of the National Institutes of Health and the policies and guidelines of the Animal Welfare Committee of the University of Texas Health Science Center at Houston (AWC, UTHSC). This study was reviewed and approved by the University Institutional Review Board (AWC approval # HSC-AWC-09-023). All surgery was performed under isoflurane anesthesia, and every effort was made to minimize suffering.

### Bacterial strains, plasmids, and growth conditions

*E. faecalis* and *Escherichia coli* strains as well as plasmids used in this study are listed in Table S1. Plasmid constructs were given pTEX numbers and their host strains corresponding TX numbers. *E. faecalis* strains were routinely grown in/on brain heart infusion (BHI) (Difco Laboratories, Detroit, MI) broth/agar or, for some experiments (see below), in tryptic soy broth supplemented with 0.25% glucose (TSBG) and, for *in vivo* experiments, in BHI supplemented with 40% horse serum (BHIS). *E. coli* strains were grown in Luria-Bertani media (Difco Laboratories). With *E. faecalis*, erythromycin (Sigma Chemical Co., St. Louis, MO) was used at 10 g/ml and, with *E. coli*, at 300 g/ml.

### Construction of deletion mutants

Single and all possible combinations of double deletions of the three *ebpABC* genes of *E. faecalis* OG1RF were created, as described previously [25]. Briefly, DNA fragments flanking one or two of the *ebp* genes to be deleted were PCR-amplified (see Table S2 for primers), joined together by cross-over PCR, and cloned into similarly digested pCJK47 [25]. After transformation into *E. coli* EC1000, the resulting plasmids (Table S1) were verified by sequencing and electroporated into OG1RF. Single crossover colonies were selected on erythromycin-containing BHI plates and then plated on MM9YEG plates supplemented with 10 mM *p*-Cl-Phe and 200 g/ml of XGal (5-bromo-4-chloro-3-indolyl-β-D-galactopyranoside) to detect colonies that had lost pCJK47 (revertants or deletion mutants). Each deletion was confirmed by PCR, sequencing, and PFGE. Of note, the last 30 bp at the 3′-end of *ebpA* and *ebpB* were left intact to not affect the ribosomal binding site of the downstream gene and 93 bp was left at the 3′-end of *ebpC* to avoid disrupting the downstream second promoter of *bps*.

### Growth curves

Growth characteristics of the OG1RF *ebpABC* deletion mutants were assessed in TSBG as described previously [26].

### Reverse transcriptase (RT)-PCR and quantitative real-time PCR (qRT-PCR)

Total RNA extraction from *E. faecalis* OG1RF and its *ebp* deletion derivatives, grown in TSBG to mid-exponential phase, and RT-PCR reactions (see Table S2 for primers) were performed as described previously [27]. A 528 bp *gdh* gene (encoding glutamate dehydrogenase) fragment was amplified as an internal control and the lack of DNA contamination in the RNA preparation was confirmed by PCR reactions in the absence of reverse transcriptase. For qRT-PCR, cDNA synthesis (SuperScript III First-Strand Synthesis System for RT-PCR, Invitrogen, Carlsbad, CA), real-time PCR amplification (MyiQ, Bio-Rad, Hercules, CA) and calculation of the fold change in relative transcript amounts by the ΔΔC_T_ method with 23S mRNA as an internal standard [28], were performed as described previously [29]. For primers, see Table S2.

### Antibodies

The previously affinity-purified polyclonal antibody preparations against EbpA, EbpB and EbpC [4] were further purified by serial affinity columns, with one of the two remaining Ebp pilus subunit proteins coupled in each to cyanogen bromide-activated Sepharose 4B [27], to remove any Ebp cross-reacting antibodies. An affinity-purified monoclonal antibody against EbpC [13] was used for western blot and IEM analyses.

### Whole-cell ELISA

Surface display of Ebp pilins by OG1RF and its *ebp* deletion derivatives was analyzed following a previously described protocol, with minor modifications [6]. In brief, bacteria were inoculated from overnight cultures and grown in TSBG broth to lag, exponential, early stationary and late stationary phases. Cells were collected by centrifugation and washed twice with phosphate-buffered saline (PBS), pH 7.4. High-binding microtiter plate wells (4HBX; Thermo Scientific, Wobum, MA) were then coated for 1 h with 100 l of cells resuspended in 50 mM carbonate-bicarbonate buffer, pH 9.6, to a cell density of OD_600_=1.0. Ebp pilin expression was detected using affinity-purified polyclonal rEbpA, rEbpB and rEbpC-specific antibodies, as described previously [6]. These antibodies showed virtually no background from their respective single and double *ebp* deletion mutants, confirming their high specificity and lack of cross-reactivity to other Ebp subunits. Total IgG antibodies purified from preimmune rabbit sera were used as an additional negative control. To confirm that cells of all strains were coated onto the microtiter wells at similar levels, we confirmed comparable binding of OG1RF and its *ebp* deletion mutants by coating and washing microtiter wells as above, followed by detection of bound cells with crystal violet staining, as in the biofilm assay below.

### Western blots of mutanolysin cell wall extracts (CW) and culture medium supernatants (Sup)

For stationary phase cells, OG1RF and its deletion mutants were inoculated from overnight cultures and grown in TSBG from an initial OD600 of 0.01 for 12 h to OD_600_^≈^1.6. For lag and exponential cells, overnight cultures were pelleted and resuspended in pre-warmed TSBG, and grown to lag (30 min, OD_600_^≈^0.2) or exponential (~2.5 h, OD_600_^≈^1.0) phases. Cell pellets and culture supernatant were then separated by centrifugation. After washing the cell pellets twice with 20 mM Tris-HCl, 10 mM MgCl_2_, 0.5 M sucrose, pH 7.0, (TMS) buffer, each cell suspension was adjusted to the same OD_600_ and then equal volumes were treated with mutanolysin in the same buffer supplemented with 1 mM PMSF and EDTA-free Complete Protease Inhibitor Cocktail (Roche Applied Science, Indianapolis, IN), followed by centrifugation to remove cells, as previously described [27].

To enable comparisons between the western blot profiles of OG1RF and *ebp* deletion mutants, all CW and Sup samples were normalized to correspond to the same original culture volume and OD600 value as that from WT OG1RF. CW and Sup fractions were TCA precipitated and washed once with acetone. Pellets were boiled in SDS-containing sample buffer, separated by 4 to 15% Tris-Glycine SDS/PAGE gradient gels (Bio-Rad, Hercules, CA) and analyzed by western blot (each sample representing equivalent amount of cells), using affinity re-purified polyclonal antibodies against rEbpA and rEbpB and monoclonal antibodies against rEbpC, followed by horseradish peroxidase-labeled secondary antibodies, as described previously [27].

### Cloning of *ebpA* into pMSP3535 and induction of *ebpA* overexpression with nisin

The complete coding sequence of *ebpA*, including its own ribosomal binding site, was amplified from genomic DNA of *E. faecalis* OG1RF with primers ebpAComF and ebpAComR (see Table S2) and cloned into the BamHI and SphI –digested shuttle vector pMSP3535 [30] under a nisin-inducible promoter. The resulting plasmid, pTEX5690, isolated from the cloning host *E. coli* XL1 Blue, was confirmed by sequencing and electroporated into *E. faecalis* OG1RFΔ*ebpA* to obtain the complemented strain TX5689. For nisin induction, overnight cultures were diluted 1:5 in fresh TSBG and grown 2h at 37 °C. These cultures were then used for inoculation of pre-warmed TSBG at OD600 of 0.01, followed by incubation at 37 °C until OD600 reached 0.5. Nisin was added to a concentration ranging from 0 to 25 ng/ml and the cultures were incubated for an additional hour at 37 °C. All above TSBG cultures were supplemented with 10µg/ml erythromycin. Nisin-induced cells were harvested and either treated with mutanolysin (see mutanolysin cell wall extracts above) or examined using IEM (see below).

### Extraction of surface-associated Ebp proteins by SDS and LiCl

Surface proteins of OG1RF and its *ebp* and sortase deletion derivatives were extracted by SDS [21] and LiCl [31] following previously described protocols, with some modifications. Briefly, bacteria were inoculated from overnight cultures and grown to exponential phase in TSBG (OD_600_^≈^1.0). Cells were harvested by centrifugation, washed twice with PBS, resuspended in 0.5 M Tris-HCl, pH 8.0, ± 0.5% SDS, and adjusted to the same OD_600_ and volume. After 1.5 h incubation at 25 °C with gentle agitation, supernatants were collected and TCA precipitated as above for mutanolysin cell wall extracts. Extraction with 1 M LiCl was performed as with SDS, except for incubation of cells for 1 h at 42 °C with 1 M LiCl.

### Immunoelectron microscopy (IEM)

To facilitate visualization of extended Ebp pili, plasmid pTEX5515, in which the positive regulator of the *ebp* pilus operon, *ebpR*, is present under a nisin-inducible promoter [12], was introduced into OG1RF *ebp* deletion derivatives by electroporation. OG1RF, OG1RF (pTEX5515) and *ebp* deletion mutants harboring pTEX5515 were grown in TSBG (pTEX5515-containing cultures were supplemented with 10 µg/ml erythromycin and 10 ng/ml nisin [11]) to exponential growth phase. Overexpression of *ebpA* in OG1RFΔ*ebpA* (pTEX5690) was induced with a series of increasing nisin concentrations as described above. Cells were then harvested by centrifugation and washed with 0.1 M NaCl. Pili from culture supernatants were recovered with ammonium sulphate precipitation as described previously [32]. Immunogold labeling was performed using anti-Ebp antibodies (1:100 dilution) or pre-immune antibodies (1:100 dilution), followed by 18 nm gold-conjugated goat anti-rabbit IgG (1:20 dilution; EbpA and EbpB staining; Jackson ImmunoResearch Laboratories, Inc., West Grove, PA) or 12 nm donkey anti-mouse IgG (1:20; EbpC staining; Jackson ImmunoResearch Laboratories), using previously described methods [32]. Samples were viewed in a Jeol 1400 transmission electron microscope.

### Biofilm formation

A biofilm density assay was performed for *E. faecalis* OG1RF and its isogenic *ebp* deletion mutants, using 96-well polystyrene plates (Becton Dickinson, Franklin Lakes, NJ) and 24 h incubation in TSBG broth, according to a previously described method [33]

### Mouse UTI model

Preparation of mice, inoculum volumes, and all other stages of the monoinfection experiment were performed according to protocols used previously in our laboratory [34,35]. Overnight cultures of *E. faecalis* OG1RF and its *ebpA, ebpB*, *ebpC* and *ebpABC* mutants, grown in BHIS, were harvested and resuspended in 0.9% saline solution to equal OD_600_ densities and CFU counts. From this, mice were inoculated via intraurethral catheterization [34] with an inoculum estimated to be ^~^ 10^5^ CFU and the inoculum was diluted and plated for actual CFU. At 48 h, mice were sacrificed, kidney pairs were excised, weighed, and homogenized in 5 ml of saline, and dilutions were plated onto BHI and bile esculin azide (BEA) agar plates supplemented with rifampin (100 g/ml) and/or fusidic acid (25 µg/ml) for CFU determination. The detection limit of bacteria in this experiment was 10^2^ CFU/g from kidney homogenates. Further, identities of the test bacteria recovered from infected organs were confirmed by colony PCR. A pre-approved protocol and guidelines by the Animal Welfare Committee of the University of Texas Health Science Center at Houston were followed throughout this study.

### Statistical analyses

Comparisons between whole-cell ELISA values of OG1RF and its *ebp* deletion mutants were analyzed by analysis of variance (ANOVA) with Bonferroni’s multiple comparison post-test. Statistical significance with qRT-PCR data was determined by the unpaired t-test. Lengths of pili produced by OG1RF and its *ebpA* mutant derivatives were compared by ANOVA with Bonferroni’s multiple comparison post-test; pairwise comparisons were performed by the unpaired t-test. The log_10_ CFU (geometric mean) of bacteria recovered from kidneys was analyzed for significance by the unpaired t test. GraphPad Prism version 4.00 (GraphPad Software, La Jolla, CA) was used for statistical analyses.

## Results

### Creation of *ebpABC* single and double deletion mutants and analysis of their growth characteristics and *ebp-bps* transcripts

**Figure 1:**
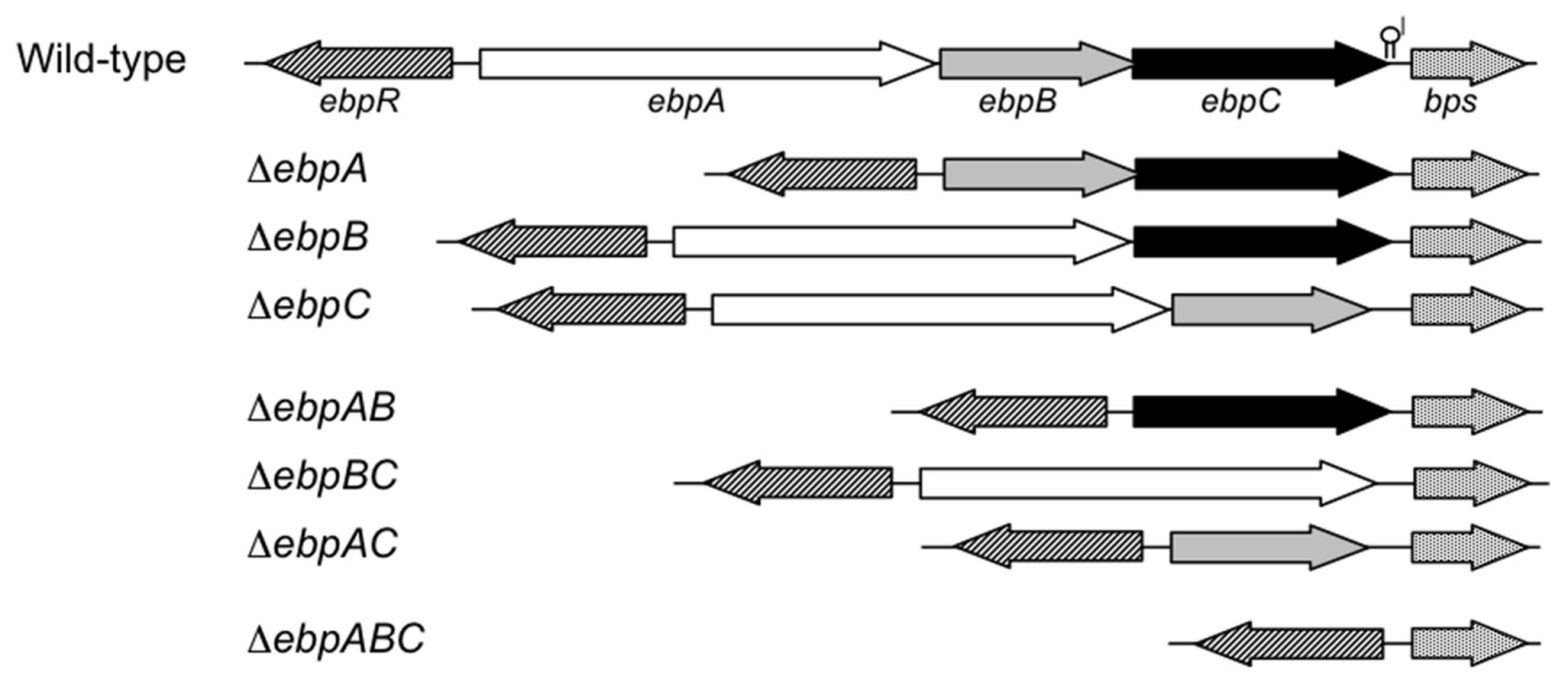
Schematic representation of the *E. faecalis* OG1RF *ebp* locus. A genetic map of the *ebp-bps* region and the gene(s) deleted from each *ebp* mutant. The previously predicted transcriptional terminator in the intergenic region between *ebpC* and *bps* is indicated with a lollipop and the lengths of the previously determined mRNA transcripts are marked by arrows above the *ebp-bps* region.

We generated six single and double in-frame deletions of the three pilin-encoding genes, *ebpA ebpB and ebpC*, of *E. faecalis* OG1RF (Figure 1). Deletions were designed to avoid affecting the ribosomal binding sites of the remaining *ebp* genes or the second promoter of the downstream sortase gene, *bps* (*srtC*), and were confirmed by sequencing. These mutants, the triple mutant OG1RF*ebpABC* [13], and wild-type (WT) OG1RF demonstrated equivalent growth kinetics by OD in TSBG (Figure S1) and comparable CFUs at different growth phases in BHI, TSBG, and BHI-S (data not shown). Subsequent RT-PCR analysis found that deletion of any one of the *ebp* genes had no appreciable effect on expression of the remaining *ebp* genes or *bps* at the transcriptional level (Figure S2). Quantitative RT-PCR confirmed that none of the deletions had a polar effect on the transcription of the downstream bps sortase gene (≤ 1.5-fold difference in *bps* transcripts of OG1RF versus any of the *ebp* deletion mutants, *P* > 0.05; data not shown)

### High molecular weight Ebp polymers are surface expressed and released into the growth medium throughout the growth cycle by *E. faecalis* OG1RF

Whole-cell ELISA analysis of WT OG1RF cells from lag, exponential, stationary and late stationary phases indicated that EbpA and EbpC are abundantly present throughout the growth cycle, albeit with gradually decreasing signals toward later stages of growth (A and B). In contrast, there was little to no EbpB detected on the cell surface in these assays (despite the clear EbpB signal from westerns of all OG1RF cell wall (CW) extracts (Figure 3).

**Figure 2:**
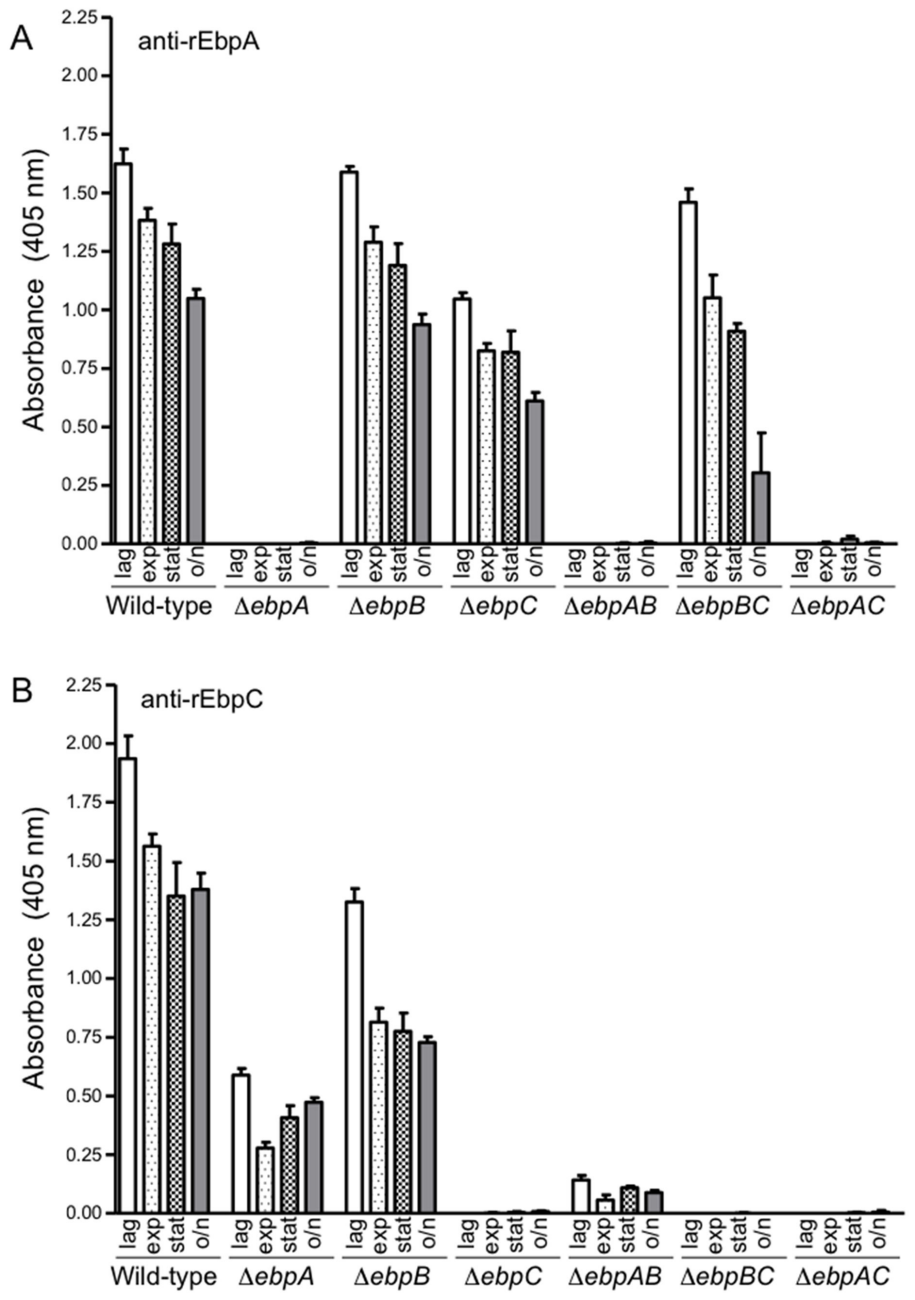
Surface display of EbpA and EbpC in different growth stages, using whole-cell ELISA. Cells were washed and adjusted to OD600 nm = 1.0 before coating onto wells. Ebp pilin expression was detected using affinity-purified and cross-absorbed polyclonal antibodies against EbpA (A) and EbpC (B). Bars represent the means of absorbance at 405 nm ± SD from four independent assays, each performed in triplicate. Mean absorbance values between WT OG1RF and its *ebp* deletion derivatives were compared using ANOVA and Bonferroni’s post-test. All mutants except lag phase Δe*bpB* with anti-EbpA were significantly reduced versus WT OG1RF from the same growth phase by post-hoc test (*P*< 0.05 to < 0.001).

**Figure 3:**
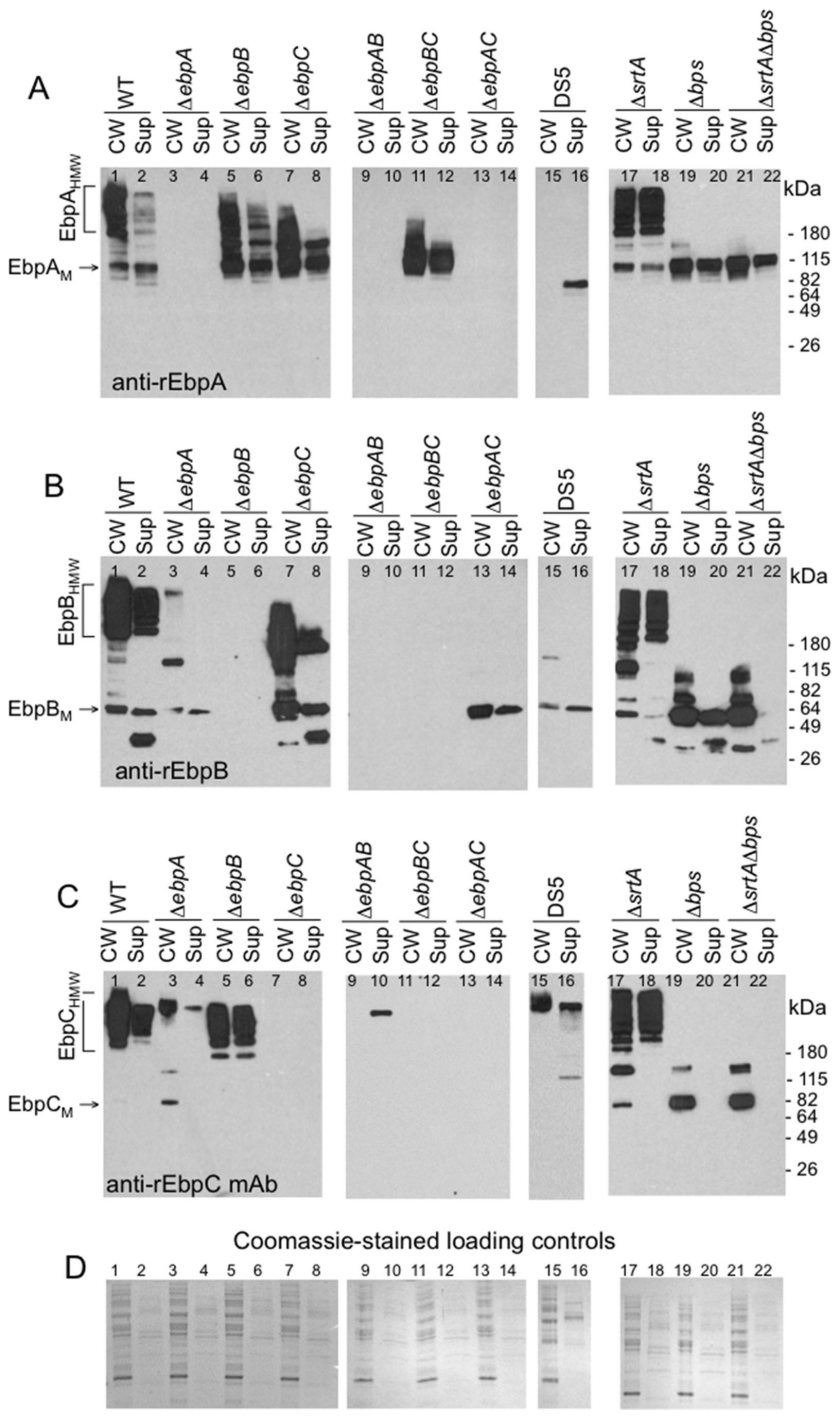
Ebp production by *E. faecalis* OG1RF, its *ebp* and sortase mutants, and strain DS5. Western blots of mutanolysin cell wall extracts (CW) and culture medium supernatants (Sup) from exponential phase cultures. (A) Immunoblot stained with polyclonal anti-EbpA antibodies. (B) Immunoblot stained with polyclonal anti-EbpB antibodies. (C) Immunoblot stained with monoclonal anti-EbpC antibodies. (D) Coomassie staining of the above CW and Sup samples; sample order as in panels A, B and C. EbpA_M_, EbpA monomer; EbpA_HMW_, high molecular weight EbpA polymers. Positions of molecular weight markers are indicated (For lag phase, see Figure S3).

Western blots of mutanolysin CW extracts from the exponential growth phase showed high molecular weight (HMW) pilus polymers that stained with antibodies to each of the three pilin proteins (Figure 3A, B and C, lane 1), consistent with our previous report of anti-EbpA/B/C stained HMW ladders from early stationary phase CW extracts [4]. Pilus polymers were also observed in the supernatants (Sup), but in reduced amounts compared to those seen with the CW samples (Figure 3A, B and C, lane 2).

Bands corresponding to the sizes of EbpA and EbpB monomers, but not EbpC monomers, were also detected in both the CW and Sup samples of WT OG1RF from all growth phases (Figure 3A, B and C, lane 1 and Figure S3, A-C, for lag phase CW extracts). Thus, our results confirm that EbpA, EbpB and EbpC are all incorporated into the pilus ladder and also released into the growth medium in all growth stages tested; however, EbpB, unlike EbpA or EbpC, seems to be inaccessible to antibodies on the OG1RF cell surface (see EM studies below).

### EbpA influences termination of pilus polymerization

Whole-cell ELISAs consistently revealed that the *ebpA* mutant had significantly reduced amounts of surface-displayed EbpC compared to WT OG1RF (median OD for *ebpA* 0.272 and for WT 1.553 with exponential phase cells, *P* < 0.001 at all growth phases) (Figure 2). Similarly, in western blots of both CW and Sup samples from the *ebpA* mutant, there was considerably less EbpB and EbpC signal detected than with WT (Figure 3B and C, lanes 3 and 4). In addition, both anti-EbpB and anti-EbpC antibodies revealed a very HMW band(s), with little to no laddering, corresponding in size to the upper region of the HMW ladder of WT OG1RF, similar to observations by Nielsen et al., using a different *ebpA* construct [5]. A band of of ca. 100 kDa was detected in the CW extract of Δ*ebpA* with both anti-EbpB and –EbpC antibodies (Figure 3B and C, lane 3), corresponding to approximately the size of the combined mass of EbpB and EbpC pilin monomers (107 kDa). This band disappeared with an additional deletion of either *ebpB* or *ebpC* in the *ebpA* mutant background (Figure 3C, lanes 9 and 13), suggesting that this band could represent a heterodimeric form of EbpB+EbpC. These data, therefore, indicate that the presence of EbpA is necessary for WT amounts of pilin polymers and for diversity in polymer size.

Among 54 *E. faecalis* strains that we recently analyzed [7], one strain (DS5) was found to have a nonsense transversion (C1967A) that generated a premature stop codon in *ebpA* (designated as ebpA*), while *ebpB* and *ebpC* were intact. We analyzed this strain for Ebp band patterns and, as expected, a single band corresponding to 2/3 the size of EbpA (ca. 69 kDa), which lacks the CW anchor region, was detected in Sup but not CW of DS5ebpA* (Figure 3A, lanes 15 and 16). As observed with *ebpA*, DS5 did not form pilus polymers of diverse sizes.

### Deletion of *ebpB* has relatively minor effects on pilus polymerization, but affects cell-wall anchoring

Western blots of the *ebpB* mutant showed EbpA and EbpC profiles that largely mimicked those of WT OG1RF except that increased amounts of pilus polymers were released into the culture medium relative to WT (Figure 3A and C, lanes 5 and 6). Additionally, we observed increased monomeric and oligomeric forms of EbpA in the *ebpB* mutant (Figure 3A, lanes 5 and 6). In whole-cell ELISA, detection of surface-displayed EbpA on the *ebpB* mutant was the same to slightly less than with WT, depending on the growth phase (Figure 2A), while a more prominent decrease in the amount of surface EbpC was seen (median OD 0.815 with exponential phase cells of *ebpB*; *P* < 0.001 versus WT OG1RF) (Figure 2B). Taken together, these data are similar to those of Nielsen et al., and confirm that EbpB is not essential for pili polymerization [5]. However, the finding of increased monomeric and dimeric forms of EbpA suggests that the absence of EbpB has some effect on polymerization, in addition to its role in attachment of pilin polymers to the cell surface; nonetheless, substantial amounts of EbpA and EbpC also remain associated with the cell surface of the *ebpB* mutant.

### Deletion of *ebpA* together with *ebpB* leads to production of very high molecular weight EbpC polymers that are released into the culture medium

Double deletion of *ebpA* and *ebpB* led to the release of a high molecular weight band of EbpC into the supernatant (Figure 3C, lane 10). In contrast, we were unable to detect polymers or monomers of EbpC in CW extracts from this mutant (Figure 3C, lane 9), which indicates that EbpC is polymerized but not anchored to the cell-wall. Consistent with the western blots, anti-EbpC signal was barely detectable in the whole-cell ELISA from the Δ*ebpAB* mutant (Figure 2B). The western blots of the Δ*ebpAB* mutant resemble those from the *ebpA* mutant in that there was only a high molecular weight band without laddering, consistent with the suggested role of EbpA in terminating pilus polymerization. Importantly, deletion of *ebpAB* also led to the release of EbpC polymers into the culture medium, similar to the partial effect seen with the Δ*ebpB* mutant, which further indicates that EbpC cannot function as a cell wall anchor subunit for Ebp pili in the absence of both minor subunits.

### Unlike the major pilin EbpC, each minor pilins is associated with the cell wall when it is the only pilin expressed

Western blots of both CW and Sup samples showed that deletion of *ebpC* resulted in a marked decrease in HMW polymers, compared to WT OG1RF (Figure 3A and B, lanes 1-2 and 7-8), with complete absence of higher MW polymers in *ebpBC* (Figure 3A, lanes 11 and 12) and *ebpAC* double mutants (Figure 3B, lanes 13 and 14). Thus, these results are consistent with previous reports on the requirement of EbpC for polymerization [4,5] and also show the apparent presence of dimers/oligomers consisting of EbpA and/or EbpB in the *ebpC* mutant (Figure 3A and B, lanes 7 and 8), similar to a recent report on heterodimerization of *C. diphtheriaeminor* pilins [32].

EbpA and EbpB monomers were observed in the westerns of both the CW extract and Sup of the *ebpC* mutant (Figure 3A and B, lanes 7 and 8), and whole-cell ELISA (Figure 2A) indicated there was abundant EbpA on the surface of *ebpC* and *ebpBC* mutants (unlike EbpB). Nielsen et al. found EbpB monomers only in the culture supernatant and not in cell lysates of their *ebpC* mutant in their western analysis [5]; this apparent difference from our detection of EbpB in CW fractions might be related to a smaller sample amount used in their western blots compared to ours. Moreover, we also found EbpB monomers in CW fractions in late log cells (Figure 3B, lane 7 and 13) as well as lag phase cells (Figure S3B, lanes 4 and 7), from both *ebpC* and *ebpAC*. These results, therefore, confirm that EbpB is associated with the cell wall when expressed as the sole Ebp subunit, similar to our observations with EbpA (Figure 3A, lanes 11 and 12), indicating that both minor pilins can be cell anchored.

### Deletion of *srtA*, encoding the housekeeping sortase, leads to increased shedding of Ebp pili into the growth medium, but does not cause major changes in pilin polymerization

Previous studies with sortase mutants of OG1RF showed that deletion of *bps* [4,5], but not insertional disruption of the *srtA* gene prevented pilin polymerization [4]. Here, we confirmed that SrtA is indeed dispensable for pilin polymerization; however, staining with antibodies against EbpA, EbpB and EbpC clearly showed increased release of HMW Ebp into the culture medium by the Δ*srtA* mutant versus WT OG1RF (Figure 3A, B and C, lanes 17 and 18), consistent with SrtA being involved in surface anchoring of polymerized pili. Nevertheless, a substantial portion of the HMW Ebp proteins was still found in the CW extracts of this mutant. Assessment of Δ*bps* and Δ*bps*Δ*srtA* mutants showed an absence of any HMW species in CW extracts and, instead, appearance of monomeric EbpA, EbpB and EbpC (and, to a lesser extent, dimeric/oligomeric forms of EbpB and/or EbpC) (Figure 3A, B and C, lanes 19-22), thus corroborating the role of Bps as the pilin-specific sortase [4,5].

### Surface-associated pili of the *srtA and ebpB* mutants can be released by a mild SDS treatment

**Figure 4:**
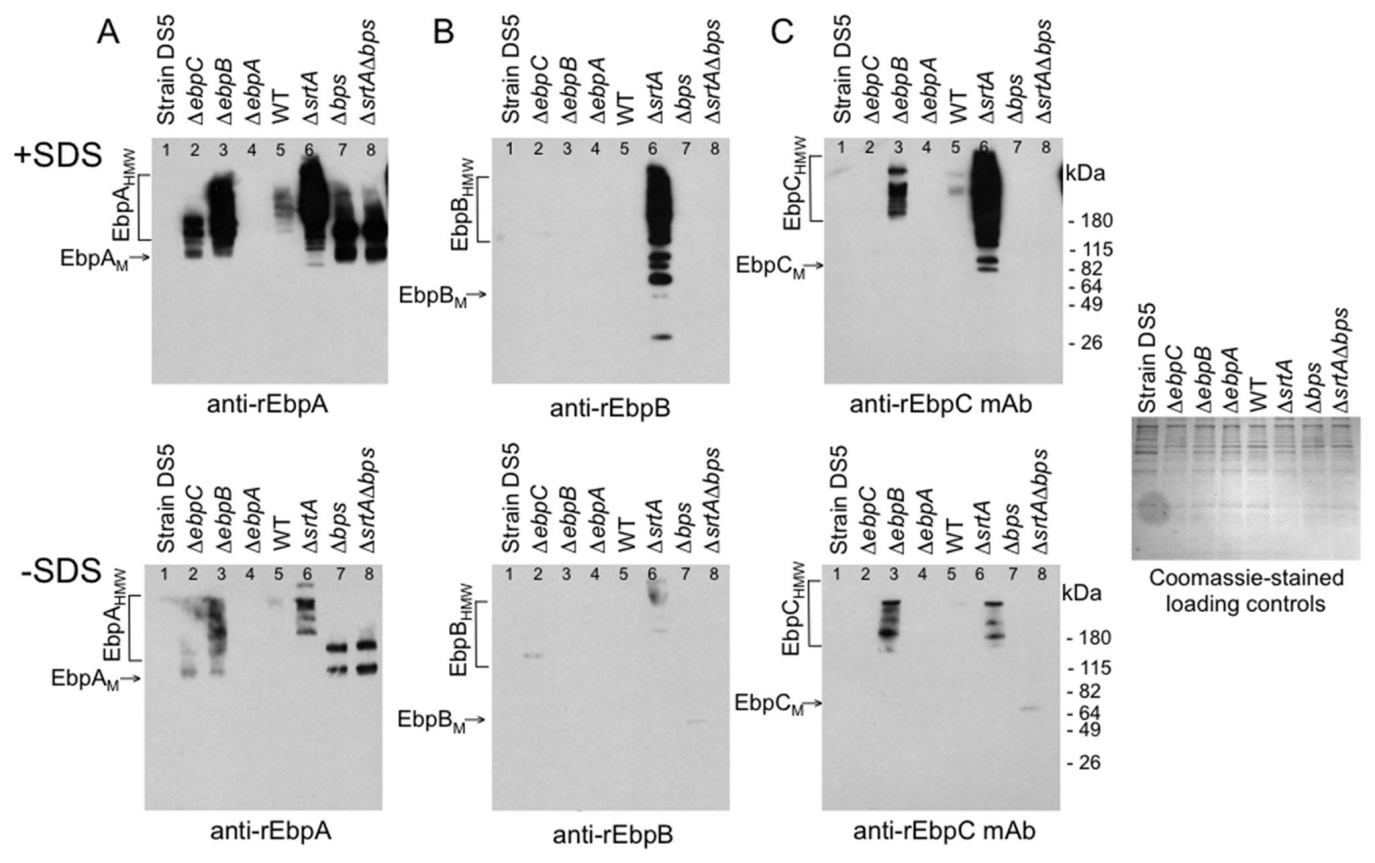
Release of surface-associated Ebp proteins from *E. faecalis* cells by a mild (0.5%) SDS treatment. Western blots of SDS extracts from exponential cells (see text for details) of strain OG1RF and its deletion derivatives and strain DS5. (A) Immunoblot stained with polyclonal anti-EbpA antibodies. (B) Immunoblot stained with polyclonal anti-EbpB antibodies. (C) Immunoblot stained with monoclonal anti-EbpC antibodies. (D) Coomassie staining of the above SDS extraction samples; sample order as in panels A, B and C. EbpA_M_, EbpA monomer; EbpA_HMW_, high molecular weight EbpA polymers. +SDS, extraction with SDS; -SDS, extraction without SDS. Positions of molecular weight markers are indicated.

Prompted by the increased, although partial, release of HMW Ebp polymers into the surrounding medium from the *ebpB* and *srtA* deletion mutants and the finding of EbpA, EbpB and EbpC in the CW fraction of the Δ*bps*Δ*srtA* mutant, we next sought to determine whether these observations could be due to surface association by interactions other than sortase-mediated covalent anchoring of pilins/pili to the peptidoglycan. For this, we first treated the cells with a detergent to release pilus subunits retained on the cell envelope by relatively weak interactions, such as those mediated through hydrophobic transmembrane domains [17,21,36], and then analyzed the samples by western blots. As seen in Figure 4, SDS treatment released only minor amounts of Ebp pilus material from OG1RF cells. In contrast, large quantities of HMW polymers containing all three pilins were released by SDS from the Δ*srtA* mutant (Figure 4, lane 6). Unlike OG1RF, therefore, the Δ*srtA* mutant has Ebp pili that are associated with cells by interactions that can be disrupted by a mild detergent treatment, in addition to the increased release of pili into the growth medium (Figure 3, lanes 17 and 18). Thus, both of these observations indicate that SrtA has a role in anchoring of Ebp pili to the cell wall. Large amounts of EbpA monomers and apparent homodimers were found after treatment of the *bps* and *srtAbps* mutants with SDS, suggesting that EbpA molecules might also be membrane-anchored, possibly through their hydrophobic tail, in the absence of *bps* and *srtA* (Figure 4A, lanes 7 and 8). A similar result was obtained by treatment with LiCl (Figure S4A), a mildly chaotropic agent commonly used for extraction of non-covalently anchored surface proteins [31,37–39]. Although EbpB and EbpC were shown above to be extractable by mutanolysin from *bps* and *srtAbps* mutants (Figure 3B and C, lanes 19 and 21), there was no detectable release of either EbpB or EbpC by SDS treatment from these two sortase mutants (Figure 4B and C, lanes 7 and 8) and only EbpB was released by treatment with LiCl (Figure S4B, lanes 7 and 8). Thus, the cell surface association of EbpB monomers in the absence of sortases resembles that of EbpA monomers except that the former seem to be more strongly associated with the cell surface, while EbpC apparently is bound to cells by an even stronger sortase-independent interaction, a yet to be characterized mechanism, which could also explain the absence of EbpC in the Sup samples of *bps* and *srtAbps* mutants (Figure 3C, lanes 20 and 22).

When the *ebpB* mutant was treated with either SDS or LiCl, a HMW ladder consisting of both EbpA and EbpC was released (Figure 4A and C lane 3; and Figure S4A and C, lane 3). Thus, deletion of *ebpB* results in an association of EbpA-C pili with cells that is SDS-extractable, as well as to increased extracellular release of pili, similar to that seen with the *srtA* mutant; this supports the hypothesis that both EbpB and SrtA have a role in cell-wall anchoring of Ebp pili. Deletion of *ebpC* led to release of monomeric and dimeric/oligomeric EbpA by SDS and LiCl (Figure 4A, lane 2 and Figure S4A, lane2) (similar to what was seen in CW extracts and Sup), although at a much lower level than from the *ebpB* mutant, suggesting that disruption of HMW Ebp polymer formation renders at least some of these EbpA molecules non-covalently linked to the cell wall even in the presence of both sortases. In contrast, no SDS-extractable EbpB was found with *ebpC* (Figure 4B lane 2) and LiCl released only a weak EbpB band (Figure S4B, lane 2). Hence, EbpB, which was released with mutanolysin, appears to be firmly cell-wall anchored in the *ebpC* mutant, similar to WT OG1RF, in agreement with the role of EbpB as a cell-wall anchoring subunit for Ebp pili.

### IEM analysis reveals EbpC along the pilus shaft, EbpA at the tip of extended pili and low amounts of EbpB on the cell surface

**Figure 5:**
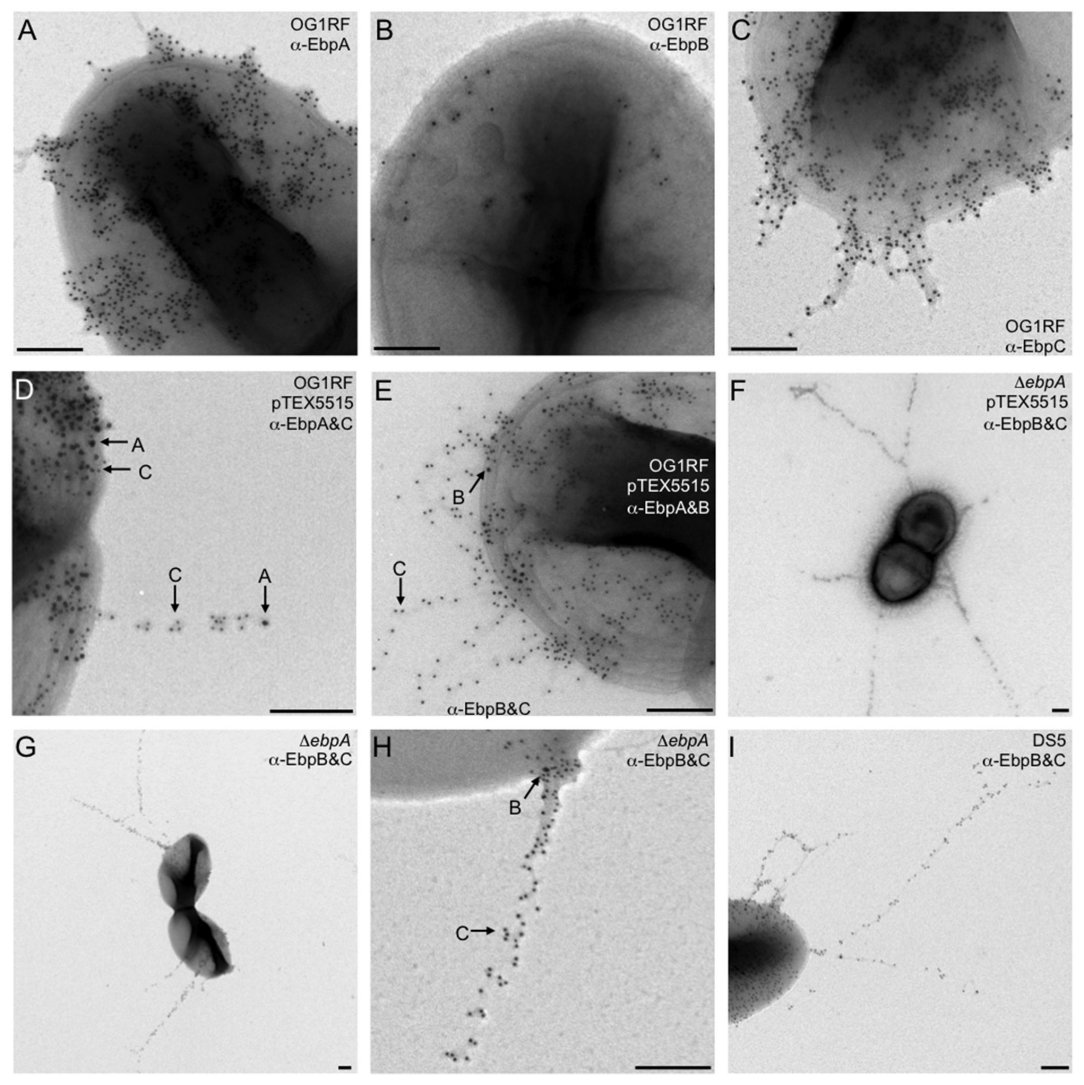
IEM analysis of pilus production by *E. faecalis* OG1RF, *ebpA* deletion mutant and strain DS5. (A) WT OG1RF stained with anti-EbpA (12 nm); (B) WT OG1RF stained with anti-EbpB (12 nm); (C) WT OG1RF stained with anti-EbpC (12 nm); (D) OG1RF (pTEX5515) stained with anti-EbpA (18 nm) and anti-EbpC (12 nm); (E) OG1RF (pTEX5515) stained with anti-EbpB (18 nm) and anti-EbpC (12 nm); (F) OG1RFΔ*ebpA* (pTEX5515) stained with anti-EbpB (18 nm) and anti-EbpC (12 nm); (G) OG1RFΔ*ebpA* stained with anti-EbpB (18 nm) and anti-EbpC (12nm); (H) OG1RFΔ*ebpA* stained with anti-EbpB (18 nm) and anti-EbpC (12nm); (I) *E. faecalis* DS5 stained with anti-EbpB (18 nm) and anti-EbpC (12 nm). Bacteria were grown in TSBG to exponential phase. In panels with antibody double labeling, examples of each stained pilin are shown by arrows. Scale bars 200 nm.

We further analyzed Ebp pili on OG1RF cells by transmission electron microscopy. The anti-EbpA antibody mostly stained material on the surface of these cells, with occasional short protrusions toward the external environment (Figure 5A). In contrast, few gold particles were seen on OG1RF with the anti-EbpB antibody staining (Figure 5B). Similar to anti-EbpA, anti-EbpC stained material was abundantly seen on the cell surface; however, anti-EbpC also detected pilus-like structures of different lengths, mostly as entangled fibrous material that occasionally could be seen extending away from the cell as distinct pilus fibers (Figure 5C), consistent with the pleomorphic pilus structures described previously [4,5]. To increase the number of pili and, thus, facilitate localization of the three different Ebp pilus subunits within the pilus filament, we next analyzed an OG1RF derivative supplemented with extra copies of *ebpR*, encoding a positive regulator of the *ebp* operon, on plasmid pTEX5515; this strain has been shown to overexpress Ebp [11]. Anti-EbpA/C double staining of OG1RF(pTEX5515) revealed large quantities of Ebp-stained material covering the cell surface on a large proportion of cells, with clearly visible distinct pili (Figure 5D). This staining indicated that EbpA is localized at the tip of extended pili (Figure 5D), while EbpC can be seen distributed along the length of the pilus shaft (Figure 5D and E). Similar to WT OG1RF, EbpA and EbpC were abundantly detected on the OG1RF(pTEX5515) cell surface; however, since Ebp pili appear to be intertwined and may have folded back onto the cell surface, it is unclear whether this cell surface EbpA and EbpC represent monomers or polymeric forms. There were very few EbpB particles detected on OG1RF(pTEX5515) (Figure 5E), similar to what is observed with WT OG1RF (Figure 5B) and consistent with the barely detectable levels of EbpB in whole-cell ELISA (see above) and flow cytometry [35]. This contrast with the strong anti-EbpB-stained bands observed in westerns of denatured cell surface extracts (as noted above in Figure 3B), suggesting that native EbpB is buried and largely inaccessible on intact cells, possibly covered by other pilus subunits or other cell surface material, at the anchor position of a pilus fiber.

We next introduced pTEX5515 into the six *ebp* deletion mutants to further assess the contribution of individual pilins to Ebp pilus production and morphology. Analysis of OG1RF*ebpA* (pTEX5515) revealed extremely long anti-EbpC-stained pili, up to several times an average cell length (Figure 5F). Although less frequent, similar very long pili were also present on the *ebpA* deletion mutant in the absence of pTEX5515, similar to recent observations by Nielsen et al. [5], demonstrating that the increased pilus length was not dependent on this *ebpR*-overexpressing plasmid (Figure 5G and H). The average length of pili produced by OG1RF*ebpA* was 960 ± 530 nm (mean ± standard deviation; median 800 nm) versus 250 ± 140 nm (median 230 nm) by WT OG1RF (*P* < 0.0001). Analysis of *E. faecalis* DS5ebpA* (a natural *ebpA* mutant, see above) demonstrated that this strain also produces much longer pili than WT OG1RF (Figure 5I). These observations are consistent with the very HMW Ebp polymers found in westerns of mutanolysin CW extracts from the OG1RFΔ*ebpA* mutant and strain DS5ebpA* (Figure 3).

**Figure 6:**
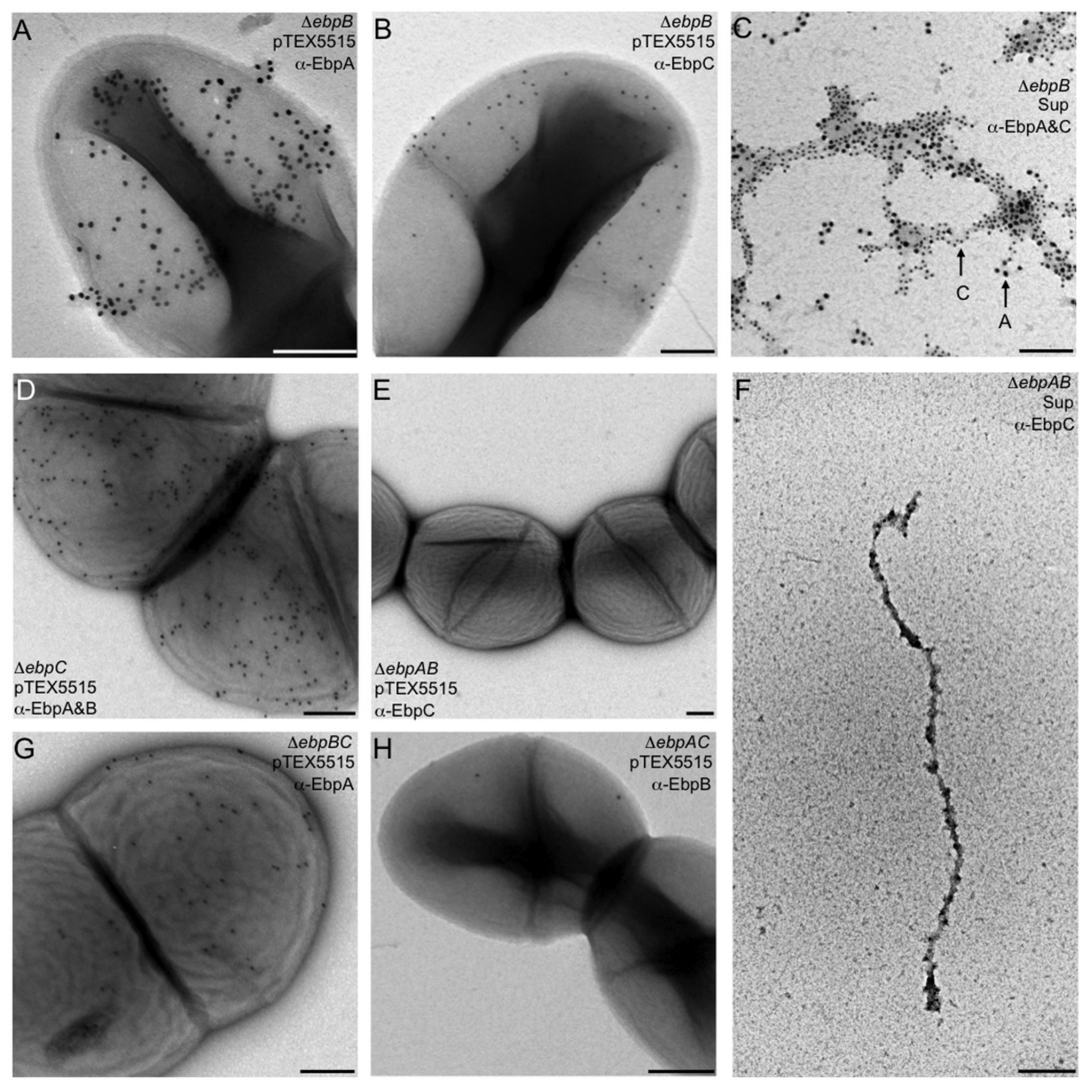
IEM analysis of pilus production by *ebp* deletion mutants of *E. faecalis* OG1RF. (A) Δ*ebpB* (pTEX5515) stained with anti-EbpA (18 nm); (B) Δ*ebpB* (pTEX5515) stained with anti-EbpC (12 nm); (C) culture supernatant of Δ*ebpB* double stained with anti-EbpA (18nm) and anti-EbpC (12 nm); (D) Δ*ebpC* (pTEX5515) stained with anti-EbpA (12 nm) and anti-EbpB (18nm); (E) Δ*ebpAB* (pTEX5515) stained with anti-EbpC (12 nm); (F) culture supernatant of Δ*ebpAB* stained with anti-EbpC (12 nm); (G) Δ*ebpBC* (pTEX5515) stained with anti-EbpA (12 nm); (H) Δ*ebpAC* (pTEX5515) stained with anti-EbpB (18 nm). All above strains were grown in TSBG to exponential phase. Scale bars 200 nm.

EbpB was detected on the cell surface of OG1RF*ebpA*(pTEX5515), OG1RF*ebpA* and DS5 in low numbers, similar to WT OG1RF, and infrequently on very long pilus fibers, similar to observations with the anchor subunit SpaB of *C. diphtheriae* [17]. Staining of the *ebpB* (pTEX5515) mutant showed both EbpA (Figure 6A) and EbpC (Figure 6B) on the cell surface but no pili could be seen, in contrast to the cell-associated anti-EbpC-stained pili recently observed on another *ebpB* deletion mutant [5]. We consider that this difference is likely due to the non-covalent surface association of the HMW polymers of *ebpB*, indicated by our western analysis of SDS and LiCl extracts described above (Figure 4 and Figure S4), leading to their detachment during processing of our IEM samples versus their sustained surface-attachment during the deep-etch IEM method used by Nielsen et al., in which the *ebpB* culture was fixed onto glass slides before antibody staining [5]. However, we did find entangled EbpA- and EbpC-stained pili upon examination of the culture supernatant of our *ebpB* mutant, in agreement with the western analyses above that showed increased amounts of EbpA/C HMW polymers in Sup of *ebpB*, thus further confirming that EbpB is not required for pilus polymerization (Figure 6C).

Analysis of *ebpC* (pTEX5515) showed EbpA on the cell surface, but not as part of extended pili, while little EbpB staining was detected (Figure 6D). Unlike Nielsen et al. [5],, we did not find EbpC associated with the surface of the Δ*ebpAB* (pTEX5515) mutant (Figure 6E), likely due to the same methodological, difference mentioned above. However, long EbpC-stained pili were seen in the culture supernatant of our *ebpAB* mutant (Figure 6F), in agreement with the lack of EbpC in the CW fraction and detection of a very HMW band(s) in the Sup of the Δ*ebpAB* mutant in westerns. These data, therefore, confirm that EbpC can be polymerized in the absence of both EbpA and EbpB and that EbpC is unlikely able to serve as a cell-wall anchoring subunit of Ebp pili. As expected from western analyses, the Δ*ebpBC* (pTEX5515) mutant expressed EbpA on the cell surface, but no pili could be seen (Figure 6G). Finally, similar to whole-cell ELISA results (but in contrast to westerns of this mutant), EbpB could be detected only occasionally on the Δ*ebpAC* (pTEX5515) mutant (Figure 6H), suggesting that neither EbpA or EbpC is responsible for EbpB’s low surface accessibility to antibodies. Taken together, these results confirm the role of EbpC as the major backbone pilin and support the role of EbpB as an anchor pilin that is buried within the cell envelope of intact cells.

### Pilus length is affected by the level of EbpA expression

**Figure 7:**
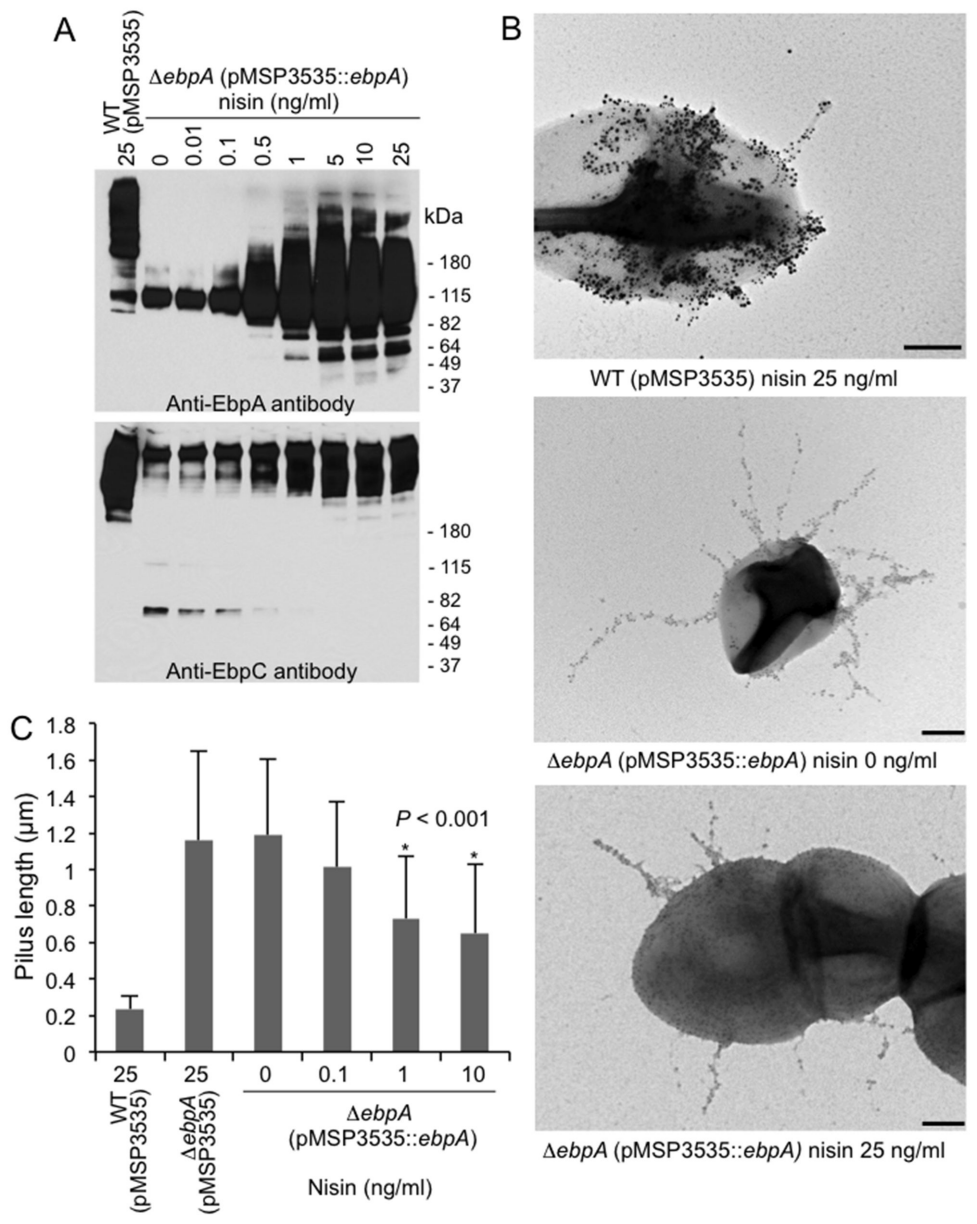
Effect of controlled overexpression of EbpA on pilus length. (A) Western blots ofmutanolysin cell wall extracts (CW) from complemented *ebpA* deletion mutant (OG1RFΔ*ebpA* (pMSP3535::*ebpA*)) grown in the presence of increasing concentrations of nisin. WT OG1RF with the empty vector (OG1RF (pMSP3535)), grown in the presence of 25 ng/ml nisin, is also shown. The membranes were probed with either anti-EbpA or anti-EbpC antibodies, as indicated. Positions of molecular weight markers are indicated. (B) IEM analysis of pilus production by cells harvested from similarly nisin-induced cultures as in panel A; stained with anti-EbpA (18 nm) and/or anti-EbpC antibodies (12 nm). Scale bars 200 nm. (C) Measurement of pilus lengths from the above IEM analysis. At least 22 pili were measured from each strain for each data point; means ± standard deviations are shown. The complemented *ebpA* deletion mutant (OG1RFΔ*ebpA* (pMSP3535::*ebpA*)) was found to produce significantly shorter pili (*P* < 0.01) after growth in the presence of 10 and 25 ng/ml nisin versus 0 ng/ml and theΔ*ebpA* empty vector control (Δ*ebpA* (pMSP3535)).

**Figure 8:**
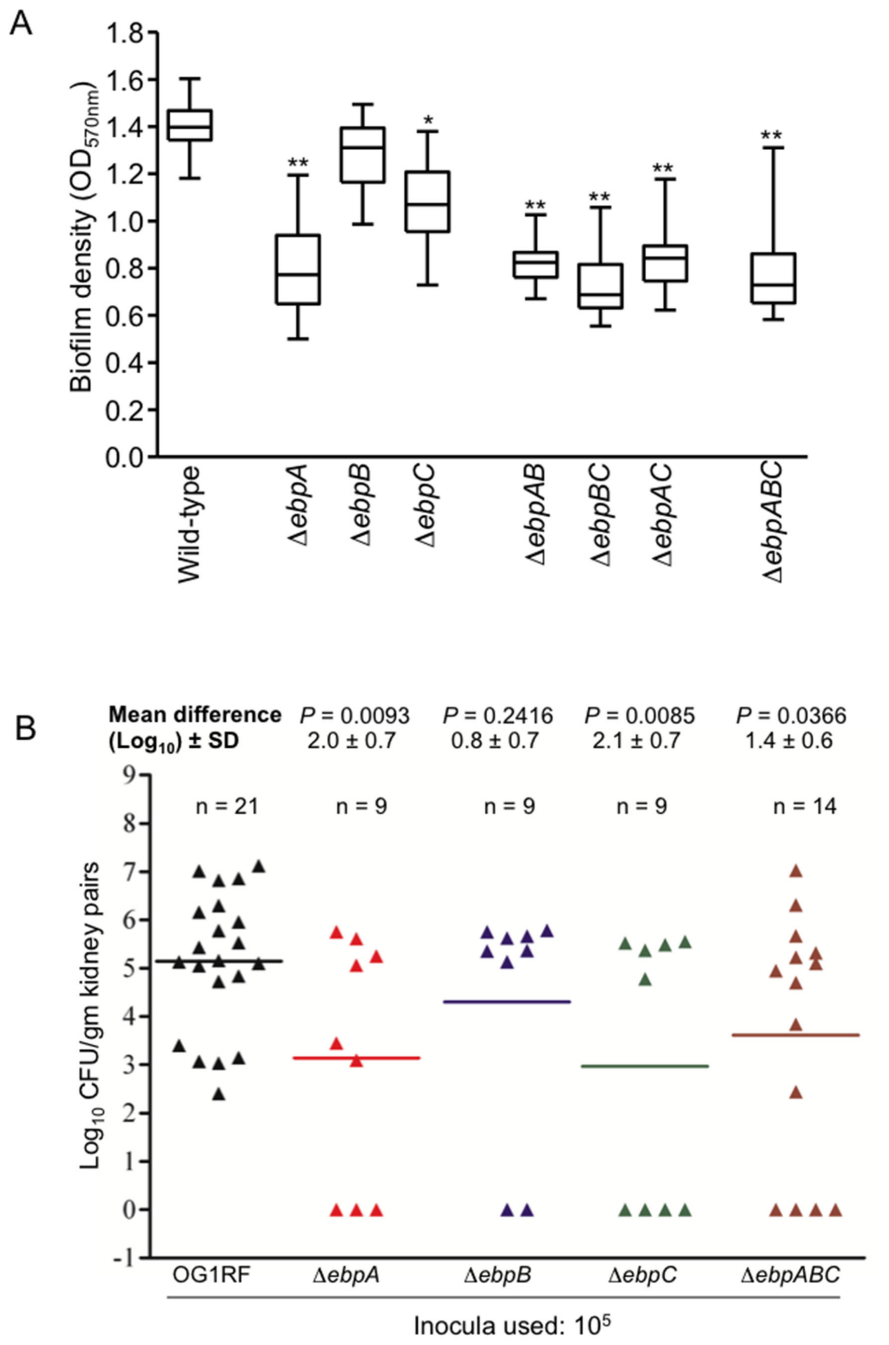
Effect of deletion of *ebp* genes in biofilm and in a murine model of UTI. (A) Comparison of biofilm formation by *E. faecalis* OG1RF and its *ebp* deletion derivates. Median OD_570_ values and interquartile ranges, with the minimum and maximum values marked by whiskers, represent combined data from three independent assays and 50 wells for each construct. Statistical analyses were performed by the Kruskall-Wallis test. *, *P*<0.001 versus WT OG1RF; **, *P*<0.001 versus WT OG1RF, Δ*ebpB* and Δ*ebpC*. (B) Monoinfection in a murine model of UTI by *E. faecalis* OG1RF and its isogenic mutants. Mice were infected with 10^5^ CFU and results are expressed as log_10_ CFU per gram from kidney-pair homogenates 48 h after transurethral challenge. A value of 1 was assigned to those kidneys with 0 CFU for statistical analyses. Black triangles represent WT OG1RF, red triangles OG1RFΔ*ebpA*, blue triangles OG1RFΔ*ebpB*, green triangles OG1RFΔ*ebpC*, and brown triangles OG1RFΔ*ebpABC*. Horizontal bars represent geometric means. The mean difference in CFU counts of *ebp* mutants versus OG1RF is given as log_10_ ± standard deviation (SD). Differences in log_10_ CFU of OG1RF versus Δ*ebpA*, Δ*ebpB*, Δ*ebpC* and Δ*ebpABC* were evaluated by the unpaired t test.

To further investigate the contribution of EbpA to pilus length, we cloned *ebpA* under a nisin-inducible promoter in the shuttle plasmid pMSP3535 for controlled overexpression in the Δ*ebpA* mutant. As seen in Figure 7A, anti-EbpA staining of mutanolysin CW extracts from the complemented *ebpA* deletion mutant (Δ*ebpA* (pMSP3535::*ebpA*)) showed gradually increasing EbpA expression with higher nisin concentrations. Although most of the expressed EbpA migrated as a monomer-size band, progressively larger amounts of higher molecular weight polymers staining with anti-EbpA were also detected as the amount of nisin was increased. Anti-EbpC staining of the same samples revealed HMW polymers of very large size in the absence of nisin, similar to what was seen with the non-complemented *ebpA* deletion mutant (Figure 7A). However, nisin induction led to gradual emergence of lower-size EbpC polymers, increasingly resembling the HMW ladder of OG1RF with the empty vector and suggesting that expression of EbpA reduces the size of EbpC polymers. Corroborating these results, IEM analysis of similarly grown Δ*ebpA* (pMSP3535::*ebpA*) cultures revealed very long anti-EbpC-stained pili in the absence of added nisin and, with increasing nisin induction, pilus length gradually decreased (Figure 7B). Measurement of pili on Δ*ebpA* (pMSP3535::*ebpA*) showed a significant decrease in average pilus length, from 1190±360 nm (no added nisin) to 650±380 nm (10 ng/ml nisin) (*P* < 0.001) (Figure 8C). Hence, these results corroborate the importance of EbpA in determining the extent of pilin elongation and suggest that the abundance of EbpA molecules plays an important role in termination of pilus polymerization.

### Deletion of *ebpA* and, to a lesser degree, *ebpC* reduces biofilm formation of *E. faecalis* OG1RF, while *ebpB* is dispensable for this phenotype

Our previous study showed reduced *in vitro* biofilm formation by polar *ebp* allelic replacement and disruption mutants versus WT OG1RF [4]. Here, we found that both the Δ*ebpABC* mutant and the Δ*ebpA* mutant showed significantly less biofilm formation compared to WT (median for Δ*ebpA*, 0.77 versus 1.40 for WT; *P*<0.001) (Figure 8A). The Δ*ebpB* mutant showed a small but non-significant reduction (median 1.31; *P*>0.05 versus WT), while deletion of *ebpC* led to a partial, and significant decrease in biofilm production (median 1.07; *P*<0.001 versus WT). Δ*ebpA* also showed significant reduction versus Δ*ebpB* and Δ*ebpC* (*P*<0.001). Consistent with these findings, all three double deletion mutants formed significantly less biofilm (*P*<0.001 versus WT). Hence, our results demonstrate that *ebpA* and, to a lesser extent, *ebpC* are both important for full biofilm production, while deletion of *ebpB*, which did not alter pilus polymerization appreciably, had little or no effect.

### Deletion of either *ebpA or ebpC* leads to attenuation of *E. faecalis* OG1RF kidney colonization in a mouse model of ascending urinary tract infection

Using a polar, non-piliated *ebpA* allelic replacement mutant (phenotypically lacking all three pilins), we have previously demonstrated that Ebp pili of OG1RF have a role in experimental mouse UTI [34]. Recently, Nielsen et al. used another mouse model designed to mimic catheter-associated UTI (CAUTI) and reported that *ebpA* and *ebpB* deletion mutants were both attenuated in bladder colonization but only the *ebpA* mutant in implant colonization, while their *ebpC* mutant was not attenuated at either infection site [5]. Here, we investigated the involvement of Ebp pili in UTI further by comparing the ability of *ebpA*, *ebpB*, *ebpC* and *ebpABC* mutants and WT OG1RF to infect kidneys in a mouse model of ascending UTI, in the absence of implanted foreign objects. Comparison of mean log_10_ CFU of bacteria recovered from the kidneys of monoinfected mice showed significant attenuation in kidney colonization when either *ebpA* (*P*=0.0093), *ebpC* (*P*=0.0085) or when the whole *ebpABC* locus (*P*=0.0366) was deleted, and slight but non-significant attenuation with *ebpB* (*P*=0.2416) versus OG1RF (Figure 8B). These data, therefore, confirm our previous results with a non-piliated mutant [34] and show that both *ebpA* and *ebpC* are required and that *ebpB* is dispensable for full virulence in our UTI model.

### Mutations disrupting gelatinase expression have relatively minor effects on Ebp pilus production

Recently, a role for the extracellular metalloprotease, gelatinase, was identified in proteolytic cleavage of the MSCRAMM Ace from the OG1RF cell surface, leading to a unique profile in which Ace disappears from the cell surface over the culture period [40]. Since a declining surface expression profile from lag to stationary phase was also observed with Ebp pili of OG1RF (Figure 2) and since Ace and the pilin subunits are predicted to have structurally similar immunoglobulin-like domains as well as cell-wall anchoring motifs, we next sought to evaluate the effect of gelatinase on Ebp cell surface expression. For this, we tested our previously created *gelE-sprE* double deletion mutant (*gelEsprE*) [40] and an *fsrB* insertional disruption mutant (*fsrB*) [41] that lack gelatinase expression. The band profiles of CW and Sup fractions from both mutants largely resembled those of WT OG1RF, except for the absence of readily detectable monomeric EbpA in either the CW or the Sup fraction of Gel^-^ mutants and the detection of only a faint very HMW species in the Sup fraction of the *gelEsprE* mutant versus a prominent HMW ladder in the Sup of WT (Figure S5). However, the lack of any noticeable reduction in the Ebp HMW intensities of cell-wall anchored ladders of either mutant versus WT OG1RF indicates lack of proteolytic cleavage of Ebp pili from the cell surface by gelatinase. Nevertheless, the absence of both cell surface-associated and released monomer-size EbpA suggests that GelE/SprE is/are important for the proper processing and/or localization of EbpA.

## Discussion

Previous studies have identified the ubiquitous pilus type of *E. faecalis*, Ebp, and demonstrated that these pili are important for *E. faecalis* infection in animal models, biofilm formation and several adherence phenotypes [4–7,34]. Here, we first sought to characterize molecular and structural aspects of the enterococcal pilus assembly process, including the subunit organization; the contribution(s) of the individual pilus subunits, EbpA, EbpB, and EbpC, to pilus polymerization and cell-wall anchoring; the minimal subunit requirement for pilus formation; and the role(s) of sortases in these processes. Our second goal was to determine the involvement of each pilus subunit in biofilm formation and virulence in a mouse model of ascending UTI.

In agreement with previous studies on the *ebp* pilus operon [4,5], western blots and IEM results unambiguously indicated EbpC as the major backbone pilin, able to polymerize into a long pilus fiber even in the absence of both EbpA and EbpB. Our data also found that pili of the *ebpAB* deletion mutant were exclusively found in the extracellular medium and no EbpC was detected on the cell surface of this mutant, indicating that EbpC is not capable of anchoring to the cell wall.

We also found that the minor pilin, EbpB, plays a major role in cell-wall anchoring of Ebp HMW polymers. First, the Δ*ebpB* mutant released more pilus polymers into the culture medium than WT, similar to observations with previously identified cell-wall anchoring subunits [17,21,22] (and also seen with the Δ*srtA* mutant). Nevertheless, considerable amounts of EbpA+C were still found in mutanolysin cell surface extracts of this mutant, possibly retained through anchoring to sortase or, possibly, to an alternative pilin anchor, such as EbpA. Second, mild treatment of Δ*ebpB* (and also Δ*srtA*, but not Δ*ebpA*) cells with SDS or LiCl both resulted in a sharp increase in released Ebp polymers compared to WT, indicating that, although EbpAC pilus polymers were present on the surface of the Δ*ebpB* mutant, they were not covalently cell-wall anchored. Hence, our data point to EbpB as the cell-wall anchoring subunit, similar to pili *C. diphtheriae* [16], *S. agalactiae* [21] and *S. pyogenes* [22]. Finally, although western blots of mutanolysin CW extracts indicate that EbpB is on the cell surface of OG1RF, several lines of evidence (whole-cell ELISA, IEM and flow cytometry [35] data) point to it being minimally accessible to antibodies when studying whole cells. Our results with EbpB resemble those with GBS150 of *S. agalactiae*, another surface-inaccessible anchor subunit whose deletion resulted in pili that were either released into the growth medium or non-covalently attached to the cell surface [21], thus suggesting a similar structural arrangement within the cell envelopes of these two organisms.

In studies of *C. diphtheriae* (SpaB) [16] and *S. pyogenes* (Spy0130) [22], deletion of the anchor pilins resulted in secreted pilus polymers that stacked at higher MW in western blots that those of the parental WT, seen also with *C. diphtheriae* as longer pili in EMs. Loss of EbpB also resulted in more polymers being present in Sup, but these pili stacked at the same or lower MW, with more apparently monomeric and dimeric EbpA on the cell surface. Thus, SpaB and Spy0130 resemble EbpB in cell-wall anchoring, but their effects on pilus polymerization and pilus length mimicked our results with EbpA (see below).

Similar to other large pilin proteins containing a von Willebrandt Factor A domain (vWF), (e.g., SpaC of *C. diphtheriae* [42], and others [43,44], our IEM studies located EbpA at the tip of the pilus fiber. Nevertheless, the majority of EbpA was located on the surface of OG1RF cells, likely representing monomer-size EbpA, as suggested in western analyses, and/or the tips of short Ebp pili curled against the cell surface. In addition, the western data also point to the possibility that EbpA may form surface-associated dimeric or low oligomeric complexes even in the absence of EbpC.

Deletion of *ebpA* diminished the overall levels of the two remaining Ebp subunits on the cell surface seen in whole-cell ELISA (Figure 2) and western blots (Figure 3), but did not alter their transcript levels. It could be also postulated that, as the tip pilin, availability of EbpA is important for efficient initiation of the sortase-mediated polymerization process, similar to the previously proposed roles of tip pilins of *Streptococcus* *suis* [45] and *Actinomyces* *oris* [46]. Importantly, IEM analyses of both *ebpA* and *ebpAB* deletion mutants demonstrated the expression of exceptionally long pili, compared to the much shorter pili of OG1RF, and westerns showed that the Ebp polymers of both mutants were stacked as HMW species that lacked the lower range of polymer sizes produced by WT OG1RF, consistent with a recent study [5]. *E. faecalis* strain DS5, a natural *ebpA* mutant that expresses a C-terminally truncated EbpA*, also produced very long surface-associated pili and its HMW profile mimicked that of the Δ*ebpA* mutant in westerns, hence confirming the effect of EbpA on pilus length and suggesting that the C-terminal end of EbpA is necessary for this process. Furthermore, controlled overexpression of *ebpA* from a nisin-inducible plasmid in the Δ*ebpA* mutant led to gradually decreasing pilus length and a broader HMW pilus ladder with a higher proportion of lower molecular weight polymers as the nisin concentration was increased; this corroborates the role of EbpA as a factor that determines pilus length and suggests that termination of pilus elongation is affected by the number of available EbpA molecules or the ratio of EbpA versus EbpB or EbpC. To our knowledge, a similar role has not been previously recognized for tip pilins of other gram-positive bacteria. In fact, while deletion of the tip pilin gene *fimQ* of *A* *. oris* also resulted in diminished initiation of pilin polymerization and fewer pili on the cell surface, these pili were shorter (unlike with Δ*ebpA*) than those of the WT parent strain [46].

Deletion of *ebpA* did not result in increased release of Ebp polymers into the culture medium nor did it lead to higher extractability of Ebp polymers by SDS or LiCl from the cell surface, as was seen with the *ebpB* and *srtA* mutants, indicating that EbpA does not act as a cell-wall anchoring subunit of pilus fibers. Thus, termination of pilus polymerization and cell-wall anchoring appear to be performed by two different Ebp pilus subunits in *E. faecalis* OG1RF. Since, however, double deletion of *ebpAB* resulted in extracellular release of EbpC pili, while a mild detergent treatment enhanced pilus release from the *ebpB* deletion mutant (but not from the *ebpA* deletion mutant), it seems likely that the presence of EbpA directly or indirectly contributes to pilus-cell surface association, at least in the absence of EbpB; however, further studies will be needed to resolve this question.

Comparison of western blots of single and double sortase mutants of OG1RF identified SrtA as the major sortase involved in anchoring of the polymerized pilus to the cell wall, indicated by (i) increased presence of pilus polymers in the culture medium of the Δ*srtA* mutant (Figure 3) and (ii) their extractability from the cell surface of this mutant by SDS and LiCl (Figures 4 and S4). These results resemble those from the Δ*ebpB* mutant and suggest that EbpB, as the pilus anchoring subunit, is the substrate for SrtA. These analyses also confirmed Bps as the pilus-specific sortase that covalently joins Ebp pilus subunits to form the pili [4,5]. Taken together, it seems plausible that pili detected in the mutanolysin CW extract of the Δ*srtA* mutant could represent pili that are still attached to the membrane-anchored Bps. Interestingly, EbpA and EbpC both have the LPXTGG cell-wall sorting motif that has previously been implicated as a preferred substrate for gram-positive pilus-associated sortases [47], whereas EbpB has LPKTN. Moreover, sequence analysis of 17 other *E. faecalis* LPXTG-like family of cell-wall anchored proteins, associated with no known pilus gene cluster in the genome of strain OG1RF and, thus, expected to be anchored by the housekeeping sortase SrtA, found none with the LPXTGG motif, while two have LPXTN and the rest (L/F/Y) PX(T/A) G(E/S/T/A) (data not shown). Consequently, it appears that (i) Bps specifically recognizes the LPXTGG motif of EbpA and EbpC, explaining why these two subunits make up the pilus tip and fiber, respectively, and that (ii) the LPKTN motif of EbpB is recognized by SrtA, guiding it then to covalently anchor this subunit to the cell wall peptidoglycan.

**Figure 9:**
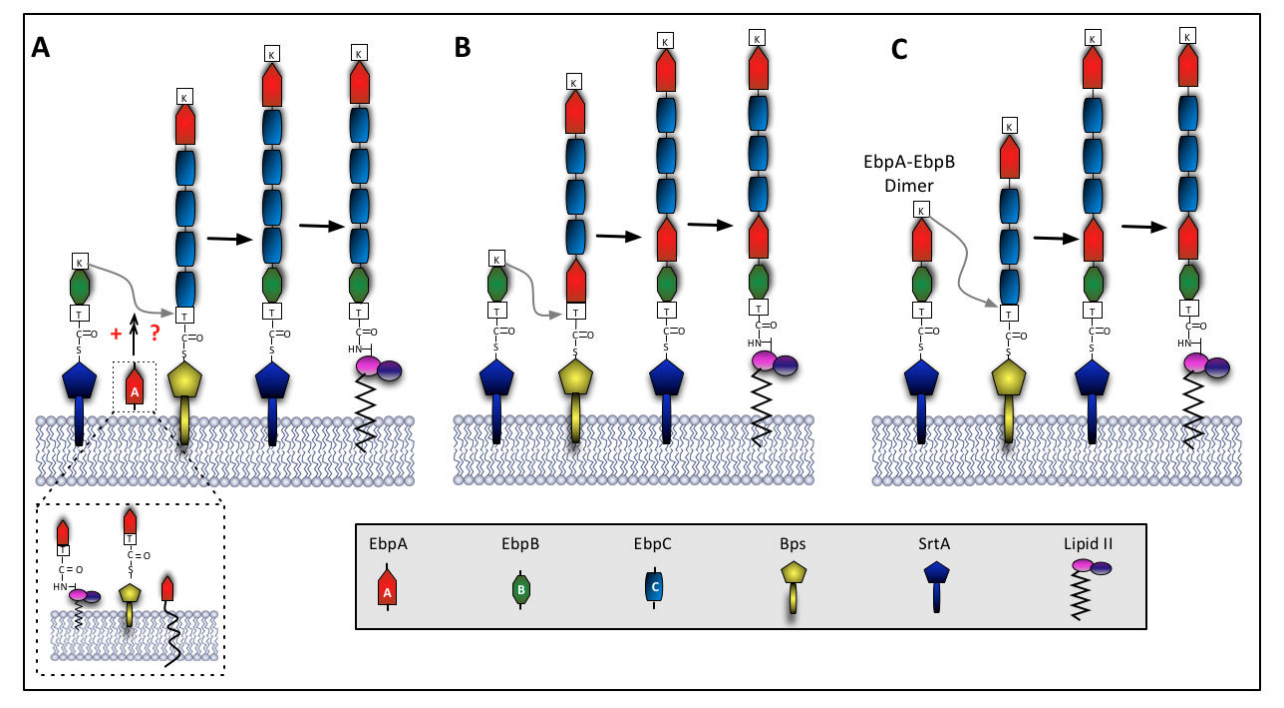
Model for pilus assembly and termination in *Enterococcus faecalis*. Ebp pilin precursors (EbpA, EbpB and EbpC) are translocated across the membrane by the Sec machinery. This is followed by a sortase-mediated reaction in which membrane-bound EbpA forms an acyl-enzyme intermediate with Bps; then EbpC is presented to this complex by another Bps molecule resulting in cross-linked subunits. Pilus polymerization continues by serial additions of EbpC subunits. We envision several possibilities for termination of Ebp pilus synthesis. (**A**) **-** The presence of cell-attached EbpA (membrane-bound, sortase-bound or anchored to the cell wall peptidoglycan) in some way stimulates the incorporation of EbpB into the pilus fiber, terminating pilus polymerization and promoting cell-wall anchoring. (**B**) **-** EbpA could be incorporated into the growing chain by Bps, which then signals addition of EbpB as the last subunit and transfer of the polymer to SrtA, which then covalently anchors the nascent pilus to the cell wall peptidoglycan. (**C**) **-**Alternatively, EbpA and EbpB could form dimers which are then incorporated into the pilus fiber, followed by transfer of the polymer to SrtA with EbpB as the last subunit of the fiber, which is then covalently anchor to the cell wall. Another possibility (not shown) is that the relative abundance of EbpA influences pilus elongation, perhaps by stabilizing the EbpB; then when EbpA is not present, as in the *ebpA* deletion mutant, EbpB becomes limited and pilus termination is less frequent.

Secretion of Ebp pilus subunits through the Sec system and their processing by either Bps or SrtA were recently shown to be spatially coupled to specific loci on the bacterial cell surface [48], similar to the ExPortal model proposed for secretion of streptococcal proteins [49]. We can extend this to a model in which membrane-bound EbpA first forms an acyl-enzyme intermediate with Bps. EbpC is then presented to this complex on another Bps molecule resulting in cleavage of the LPSTGG motif of EbpA and cross-linking of its threonine residue to a lysine within the conserved pilin motif of EbpC. Pilus synthesis then continues by serial additions of EbpC subunits to the growing pilus polymer, as proposed for *C. diphtheriae* and streptococci [16,21]. However, a question then arises as to how pilus elongation is terminated. In previous models from other pili, the cell-wall anchoring subunit serves this function [16,21], while our results identified the tip pilin EbpA as a determinant for pilus length. To reconcile this apparent paradox, we predict that pilus elongation is dependent on the relative abundance of membrane-bound pilin precursors, located within close proximity to the sortase complex (as implied by Kline et al. [48]). Perhaps when availability of EbpC is limited, this could be sensed by EbpA – possibly via EbpA-EbpC dimerization – triggering the incorporation of EbpB into an adjacent pilus base, hence terminating pilus polymerization and promoting cell-wall anchoring. Alternatively, EbpA itself could be incorporated into the growing chain by Bps, which then signals addition of EbpB as the last subunit and transfer of the polymer to SrtA, which then covalently anchors the nascent pilus to the cell wall peptidoglycan. Our previous analysis identified a well conserved pilin motif in EbpB and a less conserved pilin motif in EbpA [4]; the presence of a pilin motif in EbpA is not necessary for its location at the pilus tip. This is similar to *S. agalactiae* anchor pilins [50], but unlike other bacterial minor pilins [51,52], thus supporting a model in which EbpA as well as EbpB could be incorporated into or at the base of the pili (Figure 9).

Analysis of the contribution of each Ebp pilus subunit to phenotypic traits associated with the *ebp* locus revealed that deletion of *ebpA* reduced biofilm formation to the same level as observed with the *ebpABC* triple deletion mutant, while a less pronounced, yet significant, effect was seen with the Δ*ebpC* mutant. Thus, the extremely long EbpC- and EbpB-containing pili of the Δ*ebpA* mutant, which are fewer in number than those of OG1RF, are not capable of sustaining biofilm formation above the level seen with non-piliated cells. In contrast, the Δ*ebpC* mutant, that still expresses EbpA on its surface although not as part of pili, formed significantly more biofilm than non-piliated cells or the Δ*ebpA* mutant. Hence, these observations indicate a more important role in biofilm for EbpA than EbpC. However, since the Δ*ebpBC* mutant is more impaired than Δ*ebpC* and is similar to non-piliated cells, it is evident that the presence of EbpA with EbpB, seen as apparent heterodimers/oligomers (Figure 3), enhances biofilm formation. For WT level biofilm, however, both EbpA and EbpC are needed. Thus, it could be hypothesized that interaction with EbpB or, preferably, polymerization with EbpC extends EbpA toward the external milieu, similar to what has been suggested with *S. agalactiae* pili [44], thus enabling biofilm formation. Finally, our results with the *ebpB* deletion mutant also indicate that covalent cell-wall anchoring of Ebp pili is not needed for biofilm formation. While pilus-mediated biofilm formation has been also reported by *S. agalactiae*, *C. diphtheriae* and *A* *. oris* , there appears to be considerable variation in its mechanism, with the pilus adhesin and major backbone pilin mutants both being strongly impaired in biofilm by one *S. agalactiae* strain [44], but the cell-wall anchor and major pilin mutants resulting in attenuation by another strain of the same species [53], and the major fimbrial subunit mediating biofilm formation of *A* *. oris* [54].

In our model of ascending UTI, deletion of *ebpA* or *ebpC* resulted in equally high attenuation (^~^2 log_10_ fewer CFU) in kidney colonization; it is noteworthy that these results, in addition to those from the biofilm assay, showed only a slight, non-significant attenuation by the *ebpB* mutant versus WT. Since the ability to form biofilm is considered an important virulence-associated factor in various infections, including UTI [55,56], the involvement of EbpA and EbpC in biofilm formation could provide at least one explanation for their importance in kidney colonization during ascending UTI. EbpA was recently shown to be important also for bladder and implant colonization in a mouse model mimicking CAUTI, while deletion of *ebpC* resulted in no attenuation and deletion of *ebpB* was impaired in implant, but not bladder, colonization [5]. Also, none of these mutants significantly affected kidney colonization in this model, although an apparent trend of decreasing infectivity can be seen with both the Δ*ebpA* and Δ*ebpC* mutants versus WT [5]. This outcome is possibly due to the very low infectivity of the WT strain in kidneys in the CAUTI model (close to the limit of detection and ~4 log lower than in our ascending UTI model), which may have obscured the detection of attenuation with the *ebp* mutants. Cumulatively, the data from the above animal models support the importance of EbpA in UTI, while the effect of EbpB and EbpC appears to be more dependent on tissue type or the animal model used.

In summary, our assessment of the roles of the three structural Ebp pilus subunits and the two sortases of *E. faecalis* OG1RF in the biogenesis of Ebp pili led us to propose (i) EbpA as the tip pilin, which affects not only the number of pili but also the overall level of Ebp polymerization and pilus length; (ii) EbpB as the base subunit of pilus elongation which anchors the newly synthesized pilus polymer to the cell wall and is mostly masked on the cell surface, and (iii) the housekeeping sortase, SrtA, as responsible for cell-wall anchoring of the nascent pilus. Our data also confirm EbpC as the major pilus backbone subunit dispersed along the entire pilus shaft and the role of the *ebp* locus-associated sortase, Bps, in polymerization of pilus subunits. These results, therefore, generally agree with the pilus assembly models proposed for corynebacterial and streptococcal pili, but they also show some divergence, particularly the substantial effect of the tip pilin EbpA on pilus polymerization. We also demonstrated that EbpA and EbpC are both required for WT-level biofilm formation and for WT-level infection in kidney CFUs in an experimental model of ascending UTI, while EbpB is largely dispensable for either. Taken together, these results suggest that EbpA and EbpC, in particular, are potential targets for antibody-based approaches to deal with the increasingly antibiotic resistant and difficult to treat enterococci, further supported by their high sequence conservation and surface display among almost all *E. faecalis* strains.

## Supporting Information

Figure S1 Growth curve analysis of OG1RF and its isogenic *ebp* deletion derivatives. (A) Growth comparison between OG1RF and its *ebpB*, *ebpC*, *ebpAB*, *ebpBC* and *ebpAC* mutants. (B) Growth comparison between OG1RF and its *ebpA* mutant. Strains were grown in TSBG from an initial OD_600_ of 0.05 and samples were taken for OD_600_ readings at regular intervals.
(TIF)

Figure S2 Transcriptional analysis of the *E. faecalis* OG1RF *ebp* locus. RT-PCR of *ebp* and *bps* gene expression by OG1RF and its isogenic *ebpABC* deletion mutants. Gels on top, RT-PCR of total RNA (20 ng), isolated from mid-exponential cells and treated with DNase; gels in the middle, control reaction of the same RNA sample amplified without reverse transcriptase; gel on bottom, control reaction with genomic OG1RF DNA as template. Lane numbers correspond to the primer pairs shown in panel A. M, molecular weight marker.
(TIF)

Figure S3 Comparison of Ebp production by lag-phase cultures of *E. faecalis* OG1RF and its *ebp* mutants. Western blots of mutanolysin cell wall extracts (CW). (A) Immunoblot stained with polyclonal anti-EbpA antibodies. (B) Immunoblot stained with polyclonal anti-EbpB antibodies. (C) Immunoblot stained with monoclonal anti-EbpC antibodies. EbpA_M_, EbpA monomer; EbpA_HMW_, high molecular weight EbpA polymers. Positions of molecular weight markers are indicated by arrows.
(TIF)

Figure S4 Release of surface-associated Ebp proteins from *E. faecalis* cells by mild LiCl treatment. Western blots of LiCl extracts from exponential cells (see text for details) of strain OG1RF and its deletion derivatives and strain DS5. (A) Immunoblot stained with polyclonal anti-EbpA antibodies. (B) Immunoblot stained with polyclonal anti-EbpB antibodies. (C) Immunoblot stained with monoclonal anti-EbpC antibodies. EbpA_M_, EbpA monomer; EbpA_HMW_, high molecular weight EbpA polymers. Positions of molecular weight markers are indicated by arrows.
(TIF)

Figure S5 Comparison of Ebp production by *E. faecalis* OG1RF and its ∇*fsrB* and Δ*gelE*Δ*sprE* mutants. Western blots of mutanolysin cell wall extracts (CW) and culture medium supernatants (Sup) from exponential cultures. (A) Immunoblot stained with polyclonal anti-EbpA antibodies. (B) Immunoblot stained with polyclonal anti-EbpB antibodies. (C) Immunoblot stained with monoclonal anti-EbpC antibodies. EbpA_M_, EbpA monomer; EbpA_HMW_, high molecular weight EbpA polymers. Positions of molecular weight markers are indicated by arrows.
(TIF)

Table S1 Bacterial strains and plasmids used in this study.
(DOC)

Table S2 Oligonucleotide primers used in this study.
(DOC)
