# Supplementary figures and images for: Contribution of Individual Ebp Pilus Subunits of Enterococcus faecalis OG1RF to Pilus Biogenesis, Biofilm Formation and Urinary Tract Infection
Source: PLoS One. 2013 Jul 11;8(7):e68813. doi: 10.1371/journal.pone.0068813 (PMC3708956; doi:10.1371/journal.pone.0068813)

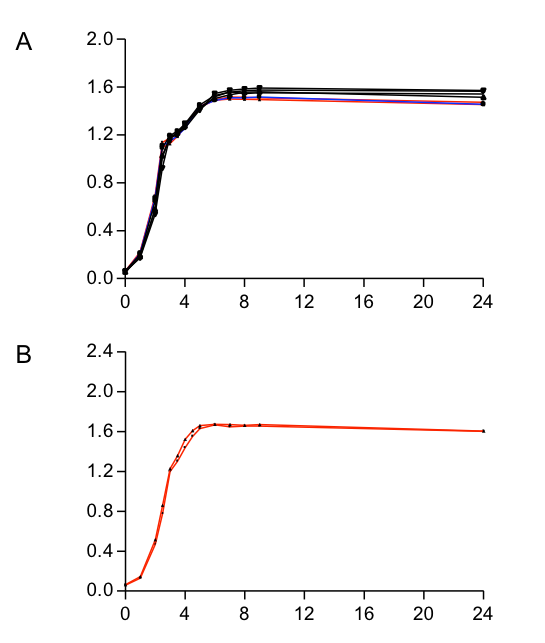

Supplement: Figure S1 — (A) Growth comparison between OG1RF and its ebpB, ebpC, ebpAB, ebpBC and ebpAC mutants. (B) Growth comparison between OG1RF and its ebpA mutant. Strains were grown in TSBG from an initial OD600 of 0.05 and samples were taken for OD600 readings at regular intervals. (TIF) [file pone.0068813.s001.tif]

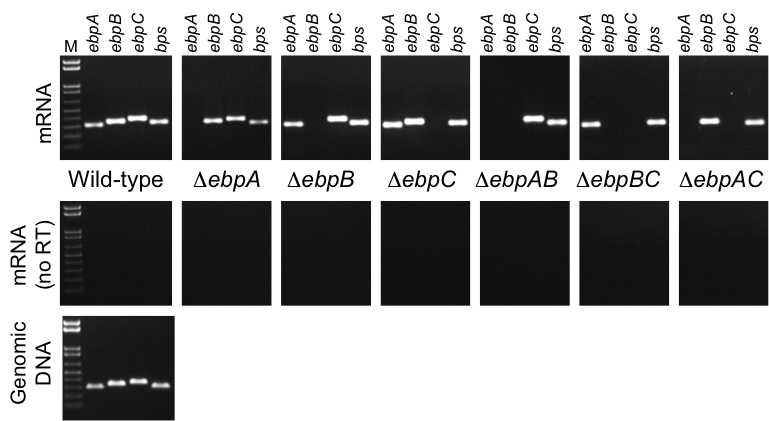

Supplement: Figure S2 — RT-PCR of ebp and bps gene expression by OG1RF and its isogenic ebpABC deletion mutants. Gels on top, RT-PCR of total RNA (20 ng), isolated from mid-exponential cells and treated with DNase; gels in the middle, control reaction of the same RNA sample amplified without reverse transcriptase; gel on bottom, control reaction with genomic OG1RF DNA as template. Lane numbers correspond to the primer pairs shown in panel A. M, molecular weight marker. (TIF) [file pone.0068813.s002.tif]

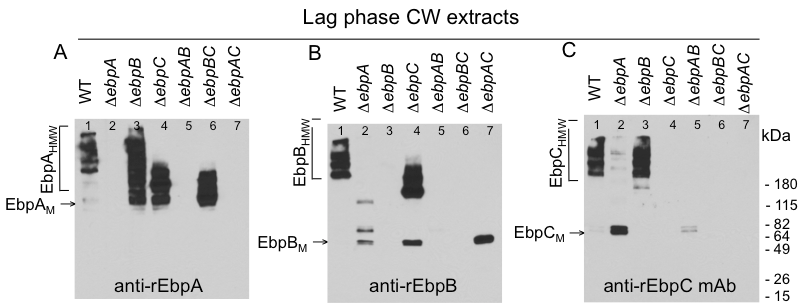

Supplement: Figure S3 — Western blots of mutanolysin cell wall extracts (CW). (A) Immunoblot stained with polyclonal anti-EbpA antibodies. (B) Immunoblot stained with polyclonal anti-EbpB antibodies. (C) Immunoblot stained with monoclonal anti-EbpC antibodies. EbpAM, EbpA monomer; EbpAHMW, high molecular weight EbpA polymers. Positions of molecular weight markers are indicated by arrows. (TIF) [file pone.0068813.s003.tif]

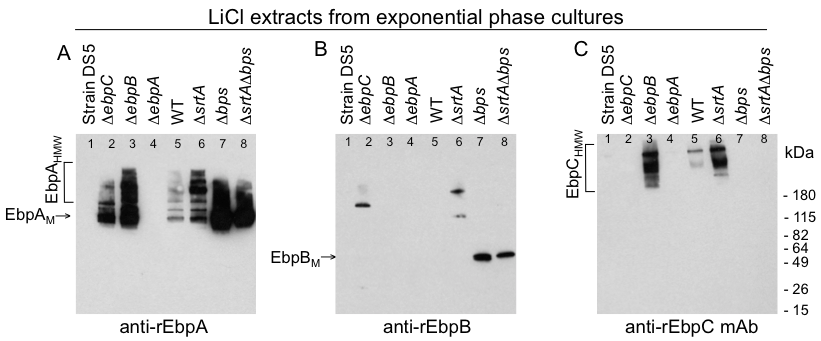

Supplement: Figure S4 — Western blots of LiCl extracts from exponential cells (see text for details) of strain OG1RF and its deletion derivatives and strain DS5. (A) Immunoblot stained with polyclonal anti-EbpA antibodies. (B) Immunoblot stained with polyclonal anti-EbpB antibodies. (C) Immunoblot stained with monoclonal anti-EbpC antibodies. EbpAM, EbpA monomer; EbpAHMW, high molecular weight EbpA polymers. Positions of molecular weight markers are indicated by arrows. (TIF) [file pone.0068813.s004.tif]

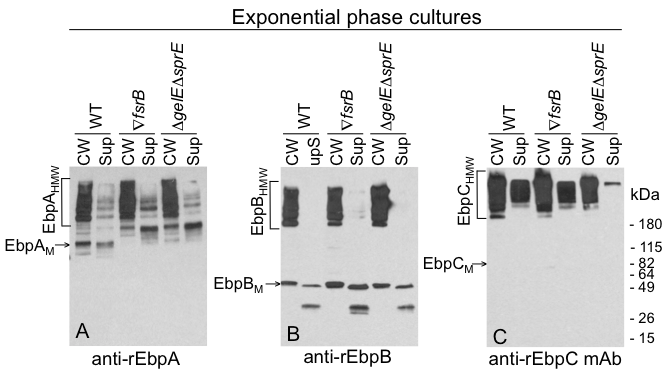

Supplement: Figure S5 — Western blots of mutanolysin cell wall extracts (CW) and culture medium supernatants (Sup) from exponential cultures. (A) Immunoblot stained with polyclonal anti-EbpA antibodies. (B) Immunoblot stained with polyclonal anti-EbpB antibodies. (C) Immunoblot stained with monoclonal anti-EbpC antibodies. EbpAM, EbpA monomer; EbpAHMW, high molecular weight EbpA polymers. Positions of molecular weight markers are indicated by arrows. (TIF) [file pone.0068813.s005.tif]
