# Supplementary material for: Contribution of Individual Ebp Pilus Subunits of Enterococcus faecalis OG1RF to Pilus Biogenesis, Biofilm Formation and Urinary Tract Infection
Source: PLoS One. 2013 Jul 11;8(7):e68813. doi: 10.1371/journal.pone.0068813 (PMC3708956; doi:10.1371/journal.pone.0068813)
Supplement: Table S1 — Bacterial strains and plasmids used in this study. (DOC) [file pone.0068813.s006.doc]

**Table S1**

| Strains/plasmids | Relevant characteristics | Reference or source |
| --- | --- | --- |
| Strains |  |  |
| *E. faecalis* |  |  |
| OG1RF | Laboratory strain; Erms, Kans, Rifr, Fusr | [57] |
| TX5608 | OG1RFΔ*ebpABC*; *ebpABC* triple deletion mutant | [13] |
| TX5620 | OG1RFΔ*ebpA*; *ebpA* single deletion mutant | This study |
| TX5621 | OG1RFΔ*ebpB*; *ebpB* single deletion mutant | This study |
| TX5622 | OG1RFΔ*ebpC*; *ebpC* single deletion mutant | This study |
| TX5623 | OG1RFΔ*ebpAB*; *ebpAB* double deletion mutant | This study |
| TX5624 | OG1RFΔ*ebpBC*; *ebpBC* double deletion mutant | This study |
| TX5625 | OG1RFΔ*ebpAC*; *ebpAC* double deletion mutant | This study |
| TX5536 | OG1RFΔ*srtA*; *srtA* single deletion mutant | [35] |
| TX5470 | OG1RFΔ*bps*; *bps* single deletion mutant | [4] |
| TX5537 | OG1RFΔ*srtA*Δ*bps*; *srtA* and *bps* double deletion mutant | [35] |
| TX5471 | OG1RFΔ*gelE*Δ*sprE; gelE* and *sprE* double deletion mutant | [40] |
| TX5241 | OG1RFÑ*fsrB*; *fsrB* insertion mutant; Gel−, Spr−, Kan^r^ | [41] |
| TX5581 | OG1RF(pTEX5515); OG1RF supplemented with *ebpR* cloned into pMSP3535; Erm^r^, Fus^r^, Rif^r^ | [12] |
| TX5682 | TX5620(pTEX5515); OG1RFΔ*ebpA* supplemented with *ebpR* cloned into pMSP3535; Erm^r^, Fus^r^, Rif^r^ | This study |
| TX5683 | TX5621(pTEX5515); OG1RFΔ*ebpB* supplemented with *ebpR* cloned into pMSP3535; Erm^r^, Fus^r^, Rif^r^ | This study |
| TX5684 | TX5622(pTEX5515); OG1RFΔ*ebpC* supplemented with *ebpR* cloned into pMSP3535; Erm^r^, Fus^r^, Rif^r^ | This study |
| TX5685 | TX5623(pTEX5515); OG1RFΔ*ebpAB* supplemented with *ebpR* cloned into pMSP3535; Erm^r^, Fus^r^, Rif^r^ | This study |
| TX5686 | TX5624(pTEX5515); OG1RFΔ*ebpBC* supplemented with *ebpR* cloned into pMSP3535; Erm^r^, Fus^r^, Rif^r^ | This study |
| TX5687 | TX5625(pTEX5515); OG1RFΔ*ebpAC* supplemented with *ebpR* cloned into pMSP3535; Erm^r^, Fus^r^, Rif^r^ | This study |
| TX5689 | TX5620(pTEX5690); OG1RFΔ*ebpA* complemented with *ebpA* cloned into pMSP3535; Erm^r^, Fus^r^, Rif^r^ | This study |
| *E. coli* |  |  |
| XL1 Blue | *E coli* host strain for routine cloning | Stratagene |
| EC1000 | *E coli* host strain for routine cloning RepA-dependent plasmids | [58] |
| TX5615 | EC1000 (pTEX5615); Kanr, Ermr | This study |
| TX5616 | EC1000 (pTEX5616); Kanr, Ermr | This study |
| TX5617 | EC1000 (pTEX5617); Kanr, Ermr | This study |
| TX5618 | EC1000 (pTEX5618); Kanr, Ermr | This study |
| TX5619 | EC1000 (pTEX5619); Kanr, Ermr | This study |
| TX5690 | XL1 Blue (pTEX5690); Ermr | This study |
| Plasmids |  |  |
| pMSP3535 | Shuttle vector for complementation, with nisin-inducible promoter; Ermr | [30] |
| pTEX5615 | Plasmid for *ebpA* deletion with flanking regions of *ebpA* cloned into pCJK47; Ermr | This study |
| pTEX5616 | Plasmid for *ebpB* deletion with flanking regions of *ebpB* cloned into pCJK47; Ermr | This study |
| pTEX5617 | Plasmid for *ebpC* deletion with flanking regions of *ebpC* cloned into pCJK47; Ermr | This study |
| pTEX5618 | Plasmid for *ebpAB* deletion with flanking regions of *ebpAB* cloned into pCJK47; Ermr | This study |
| pTEX5619 | Plasmid for *ebpBC* deletion with flanking regions of *ebpBC* cloned into pCJK47; Ermr | This study |
| pTEX5515 | pMSP3535::*ebpR;* *ebpR* including its RBS cloned under a nisin inducible promoter in pMSP3535; Erm^r^ | [12] |
| pTEX5690 | pMSP3535::*ebpA;* *ebpA* including its RBS cloned under a nisin inducible promoter in pMSP3535; Erm^r^ | This study |

Erm, erythromycin; Fus, fusidic acid; Rif, rifampin; superscript “s” designates sensitivity, “r” designates resistance; “r” is defined for enterococci as MIC >2000 for Kan; P-*pheS**, a genetic cassette for markerless exchange, containing a constitutive promoter (P) and the *pheS** allele, a counter selection marker in the presence of *p*-Cl-Phe.

**Supporting References**

57. Bourgogne A, Garsin DA, Qin X, Singh KV, Sillanpaa J, et al. (2008) Large scale variation in *Enterococcus faecalis* illustrated by the genome analysis of strain OG1RF. Genome Biol 9: R110.

58. Leenhouts K, Buist G, Bolhuis A, ten Berge A, Kiel J, et al. (1996) A general system for generating unlabelled gene replacements in bacterial chromosomes. Mol Gen Genet 253: 217-224.
