# Supplementary material for: Contribution of Individual Ebp Pilus Subunits of Enterococcus faecalis OG1RF to Pilus Biogenesis, Biofilm Formation and Urinary Tract Infection
Source: PLoS One. 2013 Jul 11;8(7):e68813. doi: 10.1371/journal.pone.0068813 (PMC3708956; doi:10.1371/journal.pone.0068813)
Supplement: Table S2 — Oligonucleotide primers used in this study. (DOC) [file pone.0068813.s007.doc]

**Table S2**

| Primer name | Sequence 5′-3′ | Function/amplicon |
| --- | --- | --- |
| Cloning |  |  |
| ebpAdel-1F | TGCTCTAGACTTCTTCTGTTAATTGTTCAATCTGTG (XbaI) | *ebpA* and *ebpAB* deletions/upstream fragment |
| ebpAdel-1R | GTCTAATAAATTTAATGTCTGTTCTCTCCTTTCTTTTATG | *ebpA* deletion/upstream fragment |
| ebpAdel-2F | AGACATTAAATTTATTAGACGACCAGAAGGGAGTGTGTAA | *ebpA* deletion/ downstream fragment |
| ebpAdel-2R | AAAACTGCAGTTTTCACTATCAACTTTGACAAAAGACT (PstI) | *ebpA* deletion/ downstream fragment |
| ebpBdel-1F | TGCTCTAGAATTTAAAAGTAATCAAAAATTCTTCCTCAG (XbaI) | *ebpB* and *ebpBC* deletion/ upstream fragment |
| ebpBdel-1R | TTTGTTTTTGTTTCATTGATTACACACTCCCTTCTGGTCG | *ebpB* deletion/ upstream fragment |
| ebpBdel-2F | ATCAATGAAACAAAAACAAAAAAGAGGAGAGAGAAAATGA | *ebpB* deletion/ downstream fragment |
| ebpBdel-2R | CCGGAATTCATTATTTGGAGCTTTTACTTCTTCTAAA (EcoRI) | *ebpB* and *ebpAB* deletion/ upstream fragment |
| ebpCdel-1F | TGCTCTAGAAAAAAGCTAGTAACCTAGTAATGATTTTGC (XbaI) | *ebpC* deletion/ upstream fragment |
| ebpCdel-1R | TTCCTTTGCCCTTCATTTTCTCTCTCCTCTTTTTTGTTTT | *ebpC* deletion/ upstream fragment |
| ebpCdel-2F | GAAAATGAAGGGCAAAGGAATCTACGTTTACTTAGGAAGT | *ebpC* deletion/ downstream fragment |
| ebpCdel-2R | AAAACTGCAGGTATGTTTAGCCTTTTCGTCATACTACTTT (PstI) | *ebpC* deletion/ downstream fragment |
| ebpABdel-1R | TTTAATGTCTGTTCTCTCCTTTCTTTTATG | *ebpAB* deletion/upstream fragment |
| ebpABdel-2F | CAAAAACAAAAAAGAGGAGAGAGAAAATGA | *ebpAB* deletion/downstream fragment |
| ebpBCdel-1R | TTTCATTGATTACACACTCCCTTCTGGTCG | *ebpBC* deletion/downstream fragment |
| ebpBCdel-2F | GGCAAAGGAATCTACGTTTACTTAGGAAGT | *ebpBC* deletion/downstream fragment |
| ebpBCdel-2R | AAAAGAATTCGTATGTTTAGCCTTTTCGTCATACTACTTT (EcoRI) | *ebpBC* deletion/ downstream fragment |
| ebpAComF | GCGGGATCCTAGAAAAGAAAGGAGAGAACAGACA (BamHI) | Cloning of *ebpA* into pMSP3535 |
| ebpAComR | GCGGCATGCTTATTTCATTGATTACACACTCCCT (SphI) | Cloning of *ebpA* into pMSP3535 |
| RT-PCR |  |  |
| Ef1091MF1 | AATGTGTTAAACCATCAAGGGAAT | RT-PCR |
| Ef1091MR1 | ACTCCTTTTTGAACTTCACCAATC | Intragenic *ebpA* [4] |
| Ef1092MF2 | CAAACAATGACTGTGTCGTATCAA | RT-PCR |
| Ef1092MR2a | AGAAGTACTGGCCATCTTTTAAAC | Intragenic *ebpB* [4] |
| Ef1093MF1 | ACAAGCTGTCCAAAGTTTAACTCC | RT-PCR |
| Ef1093MR1a | AGCCTTCGCTTTTGGAAATAACAA | Intragenic *ebpB* [4] |
| Ef1094MF2 | AATGTCCGTTTACCAATTTTTGAT | RT-PCR |
| Ef1094MR2 | GGTGTGCAAGTTAATAAAGTGACG | Intragenic *bps* [4] |
| gdhF | AGTGGCGCACTAAAAGATATGG | RT-PCR |
| gdhR | AGTTGTATTGAACCCTTGACCG | Intragenic *gdh* [4] |
| qRT-PCR |  |  |
| bps-qRT-F | TCAGGACATCGTGGTCTCCC | qRT-PCR |
| bps-qRT-R | ACTTGATAAGCAAGCGTCTTCCC | Intragenic bps |
| 23S-rRNAF | GTGATGGCGTGCCTTTTGTA | qRT-PCR |
| 23S-rRNAR | CGCCCTATTCAGACTCGCTTT | Intragenic 23S rDNA [29] |
